# Exploring the Subcellular Localization and Degradation of Spherical Nucleic Acids Using Fluorescence Lifetime Imaging Microscopy

**DOI:** 10.1021/acsnano.5c00177

**Published:** 2025-06-09

**Authors:** Steven Narum, Jiahui Zhang, Binh L. N. Vo, Joseph Nicolas Mancuso, Khalid Salaita

**Affiliations:** † Department of Biomedical Engineering, 1371 Georgia Institute of Technology and Emory University, Atlanta, Georgia 30322, United States; ‡ Department of Chemistry, Emory University, Atlanta, Georgia 30322, United States

**Keywords:** spherical nucleic acids, endocytosis, fluorescence lifetime imaging microscopy (FLIM), nanoparticles, nucleic acids, endosomal entrapment

## Abstract

Spherical nucleic acids (SNAs) are a powerful class of nucleic acids with broad applications that span from diagnostic sensors to nanoflares and gene therapeutic agents. SNAs accomplish these varied tasks by taking advantage of the programmability of nucleic acids coupled with enhanced multivalent interactions and improved cellular delivery. Nonetheless, the intracellular trafficking of SNAs remains poorly understood, as conflicting claims in the literature suggest rapid endosomal entrapment and degradation in some cases, while others suggest SNA stability and cytoplasmic escape. One of the challenges in this area is that some of the prior literature claims rely on intensity-based fluorescence measurements, which are indirect and prone to artifacts. Here, we demonstrate the use of fluorescence lifetime imaging microscopy (FLIM) as a tool to provide additional insight into the SNA intracellular fate. We specifically employ FLIM to investigate monothiol and dithiol anchored gold nanoparticle conjugates as well as phosphorothioate backbone-modified SNAs which allow us to characterize the initial stages of SNA degradation within cells. Our work shows that internalized SNAs lose up to 20% of their nucleic acids within 24 h depending on DNase II-activity and thiol-displacement in model cell lines.

## Graphical abstract

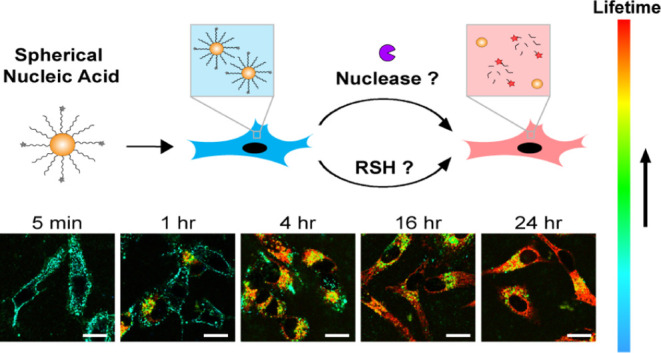

Spherical nucleic acids (SNAs) offer strategic advantages over conventional nucleic acids in biomedical applications spanning from biosensing to gene regulation (Table S1).^1–12^ Traditionally, this class of nanomedicine is composed of an inorganic nanoparticle core such as gold (AuNP) densely packed with nucleic acids, either as single or double-stranded.^13^ While double stranded (ds)­SNAs have found applications such as siRNA agents for gene regulation^14^ and as “nanoflares” to detect mRNA expression in living cells,^15–17^ single stranded (ss)­SNAs have also shown promise for immunomodulation,^18^ gene regulation,^1,2,19^ and metal ion sensing using catalytic nucleic acids.^20–22^ Additionally, the ssSNA architecture enables fine-tuning of nucleic acid density to increase hybridization efficiency with complementary strands.^23,24^ Spatial patterning of ssSNAs has been shown to enhance affinity for nucleic acid biosensors through multivalent binding interactions.^25^ Further, DNA multivalency can be used to increase specificity and selectivity in single-nucleotide polymorphism detection.^26^

SNA conjugates readily interact with cells, entering cells via endocytosis without additional carriers, which makes them ideal agents for delivering gene regulatory therapeutics (i.e., ASOs and siRNA).^27^ Depending on the size, shape, surface chemistry, and charge of the nanoparticle, the magnitude and rate of cellular uptake can be tuned.^28,29^ SNAs are also less susceptible to enzymatic degradation.^2,30^ Although the internalization of DNA-AuNP conjugates as well as their gene regulatory effects have been well demonstrated, the intracellular fate of DNA-AuNP is an important parameter and remains poorly characterized. For example, Mirkin and colleagues reported that a strong colocalization of Cy5-tagged DNA-AuNP conjugates with late endosomes can be observed even after 24 h incubation in cells, which indicates that the majority of DNA-AuNPs remain trapped in the late endosome.^30^ While innovative, nanoflares and commercial SmartFlares have been criticized due to their inability to bypass endosomal membranes, leading to doubts about mRNA colocalization or efficacy.^31^ Additionally, other works have demonstrated that nucleic acids are trapped within endosomes with a limited escape window into cell cytoplasm of up to 15 min upon endocytosis.^32,33^ This further demonstrates the need to understand how SNAs are trafficked within cells. However, determination of the integrity of DNA-AuNP conjugates is challenging as AuNPs are potent quenchers of fluorescence, which limits the analysis of DNA-AuNP colocalization.^34^

As SNAs are sequestered within endosomes, the ultimate fate is to be trafficked from early endosomes to late endosomes and finally lysosomes, where SNA degradation occurs.^35,36^ Depending on the composition of SNA, lysosomes can degrade internalized components through a multitude of enzymes such as lipases, nucleases, and proteases, which often leads to poor therapeutic performance if endosomal degradation is not overcome.^37–39^ Lysosomes are also packed with proton pumps that acidify the local environment and enable DNase II to degrade nucleic acids.^37,40–43^ This acidic environment is oxidizing and contains a higher ratio of oxidized glutathione to reduced glutathione compared to cytosolic levels, which has been implicated in diphtheria toxin-mediated endosomal escape.^44–46^

To study SNA trafficking, multicolor SNA systems have been developed to independently track DNA and nanoparticle localization in cells.^2,30^ Yet, these strategies are often reliant on covalent linkages and may underrepresent cellular nanoparticle dissociation mechanisms such as thiol-displacement of AuNP-thiol bonds by endogenous glutathione.^30,47,48^ Another study utilized correlative fluorescence and plasmonic imaging to monitor the dynamics of endosome trafficking and clustering of DNA-AuNPs, which indicated that DNA-AuNPs appear as single particles in the early stage of endocytosis, and gradually cluster during vesicular transport and maturation.^49^ The authors reported no apparent separation of fluorescence signal from Cy3-tagged DNA and plasmonic signal from AuNPs, implying colocalization of the DNA and AuNPs; however, the colocalization of signals may result from the constraint of DNA-AuNPs in intracellular vesicles, and thus does not provide direct evidence for the integrity of DNA-AuNP constructs.^49^ Ratiometric measurements of fluorophores have also been used to study SNA cellular trafficking, and while this strategy was promising, the results are inconsistent with the literature and may result from lysosomal pH-gradients or AuNP clustering, leading to confusing outcomes.^50^ SNAs become highly unstable when the nucleic acid density decreases and hence analysis of SNA integrity is critical during the initial stages of degradation in cells.^24,51–53^ The issue is that past techniques are insensitive to initial nucleic acid degradation.^30,49,50,54^ Additionally, to the best of our knowledge, comprehensive studies for SNA trafficking do not involve chemical or backbone modifications of DNA, which have been shown to be critical to further stabilize these compounds against nuclease-mediated degradation.^55,56^

Herein, we demonstrate a more direct tool, fluorescence lifetime imaging (FLIM), to monitor the initial disassembly kinetics of DNA-AuNP conjugates intracellularly and identify the factors influencing the integrity of DNA-AuNP conjugates. Our goal is to provide new insights for designing DNA-AuNP constructs that function intracellularly as therapeutics or diagnostic sensors. Unlike correlative imaging modalities, which rely on signal colocalization of the signals from the DNA and AuNP core, fluorescence lifetime depends on the local microenvironment of the fluorophore, in this case, the proximity of DNA and AuNP. The use of FLIM overcomes many of the limitations of intensity-based methods. For example, it precludes any erroneous measurements in fluorescence intensity due to changes in fluorophore density, photobleaching, and chemical degradation. Furthermore, there is no need to overlay multiple signals; hence, FLIM also circumvents complications such as fluorophore bleed-through.

Fluorescence lifetime of a fluorophore depends on its molecular environment but not its concentration. Fluorescence lifetime, τ, can be measured using timecorrelated single photon counting (TCSPC),^57^ where the sample is excited by a high frequency pulsed laser, the arrival times of the photons are recorded and fitted into a decay function to determine the fluorescence lifetime parameters, including the amplitude-weighted (τ_av amp_) and intensity-weighted (τ_av int_) averages (Scheme 1B).^58,59^ Energy transfer from a donor to an acceptor decreases the donor fluorescence lifetime; therefore, a fluorophore tagged on DNA-AuNP shows decreased fluorescence lifetime due to nanometal surface energy transfer (NSET), which results from proximity quenching by the AuNP surface. By quantifying the fluorescence lifetime of a fluorophore tagged on DNA that is attached to AuNPs, we aimed to map the spatial and temporal dynamics of DNA-AuNP conjugate disassembly, as well as the role of nuclease-caused degradation and detachment of DNA from AuNP on the disassembly of the conjugates.

**Scheme 1:**
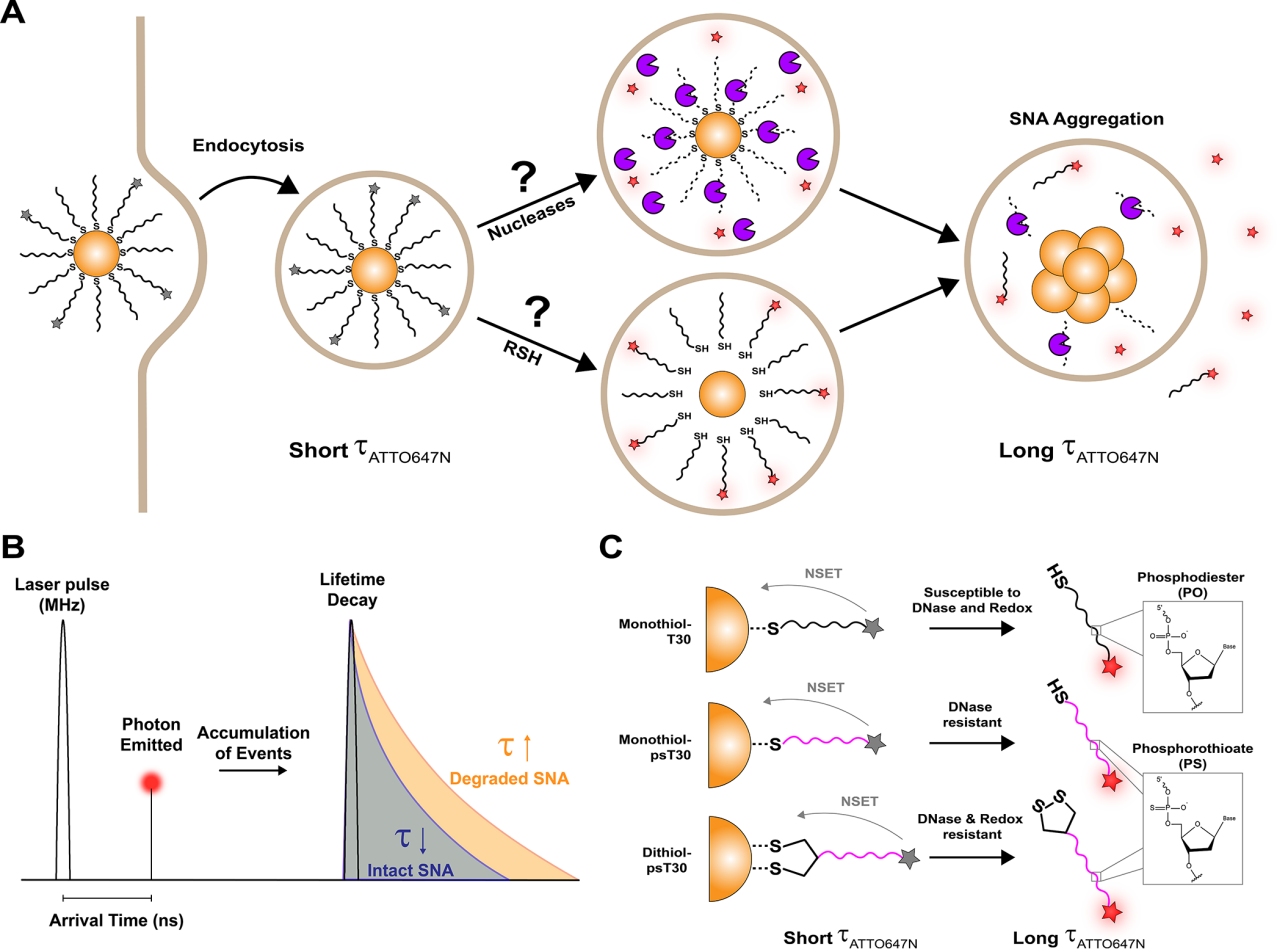
Understanding the Degradation Pathway of SNAs^a^ ^b^ ^c^

a An SNA is functionalized with a dense layer of DNA, which is conjugated to an Atto647N dye molecule. The dye is quenched by the AuNP core through NSET, leading to short fluorescence lifetimes. The SNA enters the cell via endocytosis, where the DNA shell is ultimately degraded through nucleases or dissociated through thiol–disulfide exchange, although the predominant mechanism remains unknown. Once DNA has detached from the AuNP core, the SNA aggregates, while the Atto647N dye is unquenched, leading to longer fluorescence lifetimes.
b A rapidly pulsed MHz laser excites Atto647N dye, which emits a photon after a short ns period. FLIM measures the accumulation of arrival times to create a lifetime decay plot, which can be short (blue) or long (red) depending on the quenching state of the dye.
c Three SNA constructs containing phosphodiester (PO, black) or phosphorothioate (PS, pink) backbones, and a monothiol or dithiol modification are used in this study to distinguish the SNA cellular dissociation mechanism. The PO backbone is readily degraded by DNases (DNase I or II), while the PS backbone is resistant to these. Additionally, the monothiol modification is more prone to thiol–disulfide exchange compared to the dithiol modification.

## Results and Discussion

After internalization of DNA-AuNP constructs in cells, DNA-AuNPs are trafficked through the endolysosomal pathway, which is known for its acidic and degradative environment. To preclude the influence of pH on the fluorescence lifetime of the fluorophore, we chose ATTO647N as the fluorescence tag, whose fluorescence lifetime only shows slight changes in PBS with different pH levels. Specifically, the fluorescence lifetime of ATTO647N-DNA in PBS with pH 7.4, 4.6, and 3.4 were measured to be 4.6, 4.1, and 3.7 ns (τ_av amp_) and 4.7, 4.2, and 3.9 ns (τ_av int_) calculated from biexponential reconvolution fitting of the FLIM decay curve (Figure S1). This minimal lifetime decrease in the endolysosomal pH range (7.5–4.5) confirms that ATTO647N is a suitable dye to study nanoparticle internalization.

Next, we sought to demonstrate that the fluorescence lifetime measurement can distinguish between soluble DNA and DNA attached to AuNPs. Therefore, we attached thiolated T30 strands tagged with ATTO647N (1:10) on AuNP and measured the fluorescence lifetime of the soluble T30 strand and T30-AuNP conjugates in PBS (Figure 1A–C). As expected, ATTO647N-T30-AuNPs exhibited much faster decay in the FLIM decay curve and shorter fluorescence lifetime (τ_av amp_ = 0.8 ns, τ_av int_ = 1.6 ns) compared to the ATTO647N-T30 strand (τ_av amp_ = 3.3 ns, τ_av int_ = 3.5 ns) due to NSET (Figures 1B,C and S2A). We chose to use a 1:10 labeling ratio of ATTO647N-DNA strands to unlabeled DNA to minimize the effect of homoFRET as well as prevent unintended changes in nuclease degradation or stability (Figure S8F).

**Figure 1:**
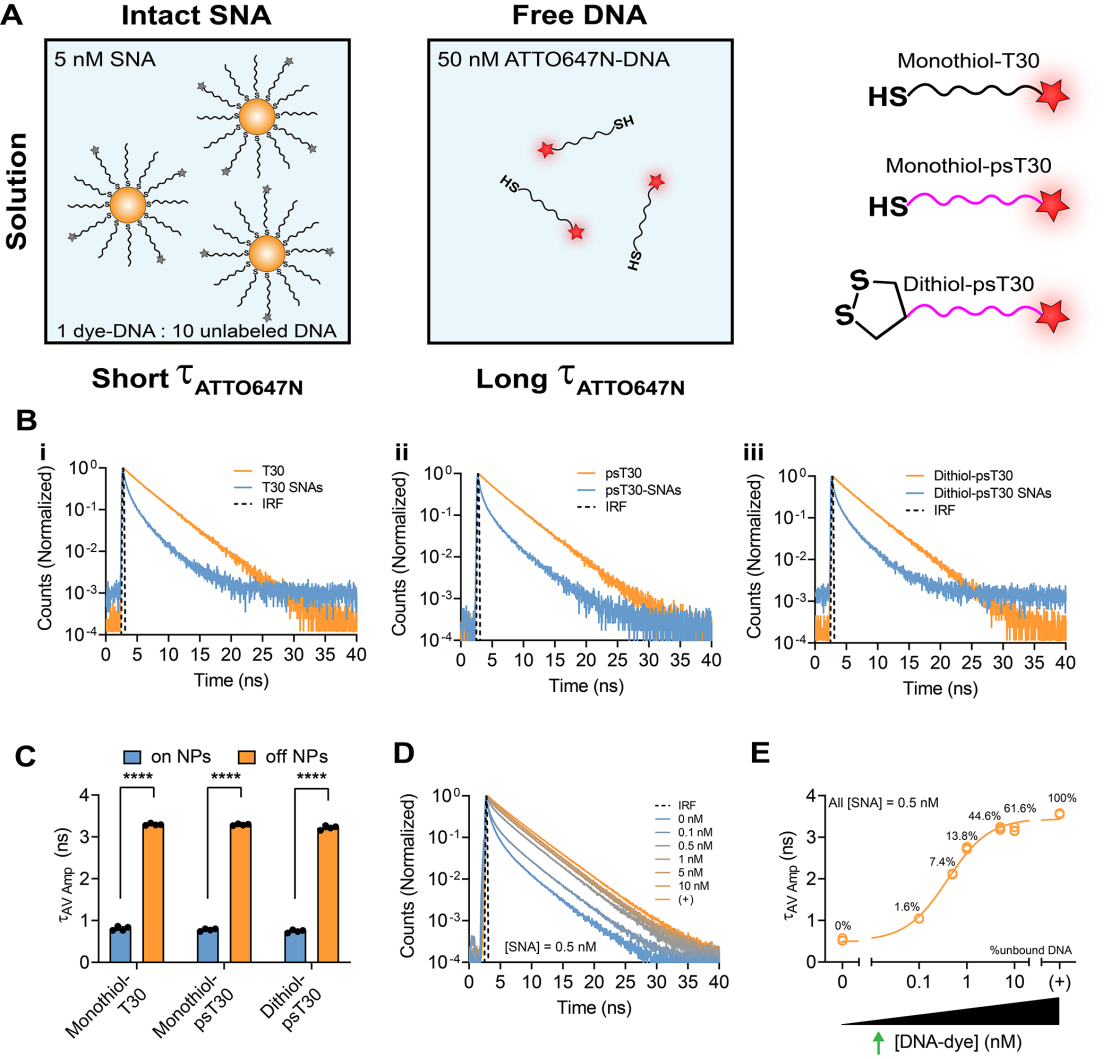
FLIM measurements reveal distinct lifetime states dependent on NP proximity in solution. **A.** Schematic showing the solution-based experimental design for intact SNAs (5 nM, left) and free ATTO647N-DNA (50 nM, right). SNA and DNA measurements were conducted for all three DNA structures shown (monothiol-T30, monothiol-psT30, and dithiol-psT30). **B.** Normalized decay curves of 50 nM: **i.** monothiol-T30 DNA, **ii.** monothiol-psT30 DNA, and **iii.** dithiol psT30 DNA in PBS as soluble ATTO647N-DNA (orange) or bound to SNA (blue). The instrument response function (IRF) is colored black for reference. **C**. Plot showing the average amplitude lifetime for all three constructs when bound to NP (blue) or off NP (orange). **D**. Normalized decay curves for a titration of unbound ATTO647N-DNA (0–10 nM) added to a 0.5 nM DNA-AuNP solution in 1× PBS. The positive control (+) is 50 nM DNA only. **E.** Plot showing the average amplitude lifetime for a titration of unbound ATTO647N-DNA (0–10 nM) to 0.5 nM DNA-AuNP (∼6 nM ATTO647N-DNA) in 1× PBS. The values associated with each data point indicate the percentage of nucleic acid that is soluble which provides a calibration of DNA %unbound. Data were fit to a sigmoidal function. The positive control (+) is ATTO647N-DNA without AuNP. Statistics were conducted using student *t* tests with *p* values reported as **** (*p* < 0.0001). Experiments were measured in triplicate at RT.

As shown in Scheme 1A, there are potentially two mechanisms that may contribute to the disassembly of DNA-AuNP conjugates intracellularly: (1) breakage of the Au-thiol bond due to intracellular reducing agents; and (2) DNA degradation caused by intracellular nucleases. To study the role of nucleases and the reducing environment on the integrity of DNA-AuNP conjugates, we also prepared two types of DNA-AuNPs conjugates. One is T30-AuNP conjugates with a PS-modified backbone (monothiol-psT30-NP), which offers nuclease resistance and increases the stability of DNA (Table S2). The second employed bidentate Au-thiol bonds instead of a monothiol-Au bond, in addition to PS modification (dithiol-psT30-NP), to increase stability against reducing agents as well as nuclease resistance (Scheme 1C). Previous studies have reported that DNA conjugation to AuNP surface with cyclic disulfide was more stable compared to a monothiol group under the treatment of competing thiols, such as mercaptohexanol and dithiothreitol (DTT).^60^ As shown in Figure 1C, monothiol-psT30-NPs and dithiol-psT30-NPs tagged with ATTO647N also showed reduced fluorescence lifetimes compared to their soluble linear counterparts, similar to monothiol-T30-NPs.

Next, we investigated the lifetime of binary mixtures of ATTO647N-tagged DNA-NP and soluble ATTO647N-DNA. Here, we measured the fluorescence lifetime of mixtures of 0.5 nM monothiol-T30-NP with different concentrations of soluble T30 strands. The result showed an increased fluorescence lifetime with the increase of soluble T30 strands titrated in the solution (Figures 1D,E and S2B). This confirms that FLIM could be used to quantify the fraction of dissociated fluorophore in a mixture (Figure S9A). Note that 0.5 nM monothiol-T30-NP presents a concentration of 6 nM ATTO647N-DNA (Figure S8), and when the ratio of ATTO647N on and off AuNP is 1:1, the τ_av amp_ of the mixture approaches that of the soluble strand because the signal from the soluble DNA dominates the average lifetime (Figure 1D,E). This calibration indicates that FLIM is highly sensitive to the very initial stage of DNA dissociation from the SNA when less than 25% of the nucleic acid is released from the SNA. Conveniently, loss of 25% of DNA density on an AuNP leads to AuNP aggregation and loss of SNA function, hence underscoring the power of FLIM in characterizing the most relevant stages of SNA degradation.

Next, we tested the chemical stability of bidentate thiol-Au bond compared to the monothiol-Au bond (Figure 2A). We incubated 0.5 nM monothiol-psT30-NPs or dithiol-psT30-NPs with 100 μM DTT for 5 and 30 min at 37 °C and measured fluorescence lifetime. The fluorescence lifetime of ATTO647N on both monothiol-psT30-NPs and dithiol-psT30-NPs increased after 5 min of incubation, indicating the release of ATTO647N-tagged DNA (Figures 2B and S3A-C). However, the increase in fluorescence lifetime of dithiol-psT30-NPs group is less than monothiol-psT30-NPs. This confirms the higher stability of the bidentate thiol-Au bond compared to the monothiol-Au bond. The fluorescence lifetime of both samples continued to increase after 30 min of incubation, and still the dithiol-psT30-NPs group exhibited a shorter fluorescence lifetime than monothiol-psT30-NPs (Figures 2B and S3A-C). To determine the effectiveness of DNA phosphorothioate (PS) modification to resist nuclease degradation, we incubated 0.5 nM monothiol-T30-NPs (phosphodiester linkages) or monothiol-psT30 (PS linkages) with 5 units of either DNase I or DNase II to monitor nucleic acid degradation over a 24 h period (Figure 2C). Previous reports have shown that DNase I activity requires divalent cations such as Mg^2+^ or Mn^2+^,^61^ while DNase II is most active in acidic pH (∼5.0) independent of divalent cations, so we used optimized buffers for each DNase, respectively.^42^ We found that monothiol-T30-NPs were more prone to both DNase I and II degradation compared to monothiol-psT30-NPs as indicated by a rapid increase in fluorescent lifetime and nearly complete dissociation in only 2 h (Figure 2D,E and S3D,E). As a control, we confirmed that the DNA-AuNP constructs remained stable in solution for 24 h in the absence of reducing agents or nucleases (Figure S3F,G).

**Figure 2:**
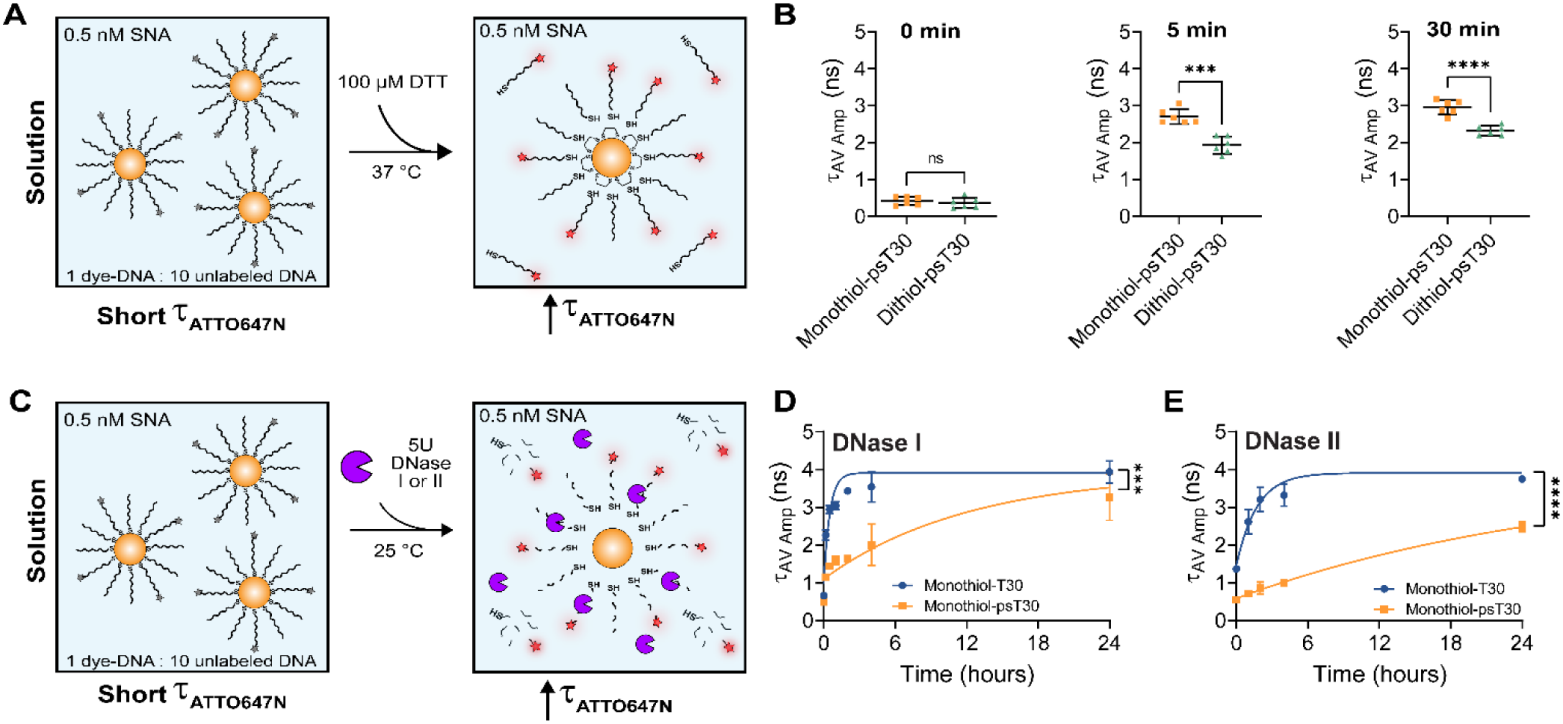
DNA modifications enhance the SNA stability against reducing agents and nucleases. **A.** Schematic showing SNA dissociation with the addition of 100 μM DTT in solution. ATTO647N dyes are quenched via NSET when proximal to the AuNP core (short lifetimes) and unquenched with thiol displacement due to DTT (long lifetimes). **B.** Plots showing the average amplitude lifetime for 0.5 nM monothiol-psT30 and dithiol-psT30 SNA constructs after 0, 5, and 30 min incubations at RT in 1× PBS. **C.** Schematic showing nuclease-mediated degradation (DNase I or II) of SNAs, leading to longer ATTO647N lifetimes in solution. **D.** Plot showing average amplitude lifetimes for 0.5 nM monothiol-T30 and monothiol-psT30 SNA constructs after treatment with 5U DNase I over a 24 h period in DNase I optimized buffer (10 mM Tris-HCl, 2.5 mM MgCl_2_, 0.1 mM CaCl_2_, pH 7.5). **E.** Plot showing average amplitude lifetimes for 0.5 nM monothiol-T30 and monothiol-psT30 SNA constructs after treatment with 5U DNase II over a 24 h period in DNase II optimized buffer (1× UB4 buffer, 117 mM NaCl, pH 5.0). Statistics were conducted using an unpaired student’s *t* test (**B**) or extra sum-of-squares *F* test (**D**, **E**) with *p* values reported as ns (*p* > 0.05), *** (*p* < 0.001), and **** (*p* < 0.0001). Measurements were conducted in at least triplicate.

After FLIM characterization of the DNA-AuNP conjugates in buffer, we moved on to monitoring their intracellular disassembly to elucidate the role of nucleases and reducing environment on their integrity. For this purpose, we pulse-treated HeLa cells with 5 nM of DNA-AuNPs for 5 min before washing the cells to remove excess AuNPs from the solution (Figure 3A). We chose a pulse-treatment strategy to minimize signal dilution compared to sustained treatment, as cells continuously recycle surface receptors,^62^ which would complicate the analysis of DNA-AuNP dissociation. We found that DNA-AuNPs rapidly enter cells as a substantial amount of cell-associated fluorescence was measured, despite the brief 5 min incubation (Figure 3B). Visually, it is clear that all three constructs exhibit short lifetimes (blue-teal color) at early time points with increasing lifetimes throughout the 24 h timelapse (Figure 3B). Additionally, the signal is punctate and sparse at early time points, which indicates endosomal entrapment rather than cytosolic delivery. By the 24 h time point, all DNA-AuNP constructs show high levels of degradation as indicated by long (red color) lifetimes as well as higher confluency, suggesting cytosolic distribution (Figure 3B). By analyzing the kinetic profile of the average amplitude lifetimes, dithiol-psT30-NPs are the most stable group (*t*_1/2_ = 31.6 h^–1^), followed by monothiol-psT30-NPs (*t*_1/2_ = 26.2 h^–1^), with monothiol-T30-NPs (*t*_1/2_ = 11.4 h^–1^) being the most prone to cellular dissociation (Figures 3D and S4A). Additionally, the PS-modified NPs are significantly more stable than the phosphodiester (PO) NPs, which implicates nuclease susceptibility as a leading factor in cellular dissociation. To further explore this idea, we chose RAW264.7 macrophages as macrophages have high expression levels of nucleases compared to epithelial cells.^63^ Following a similar treatment strategy as before, we also found punctate signals and short lifetimes for all three NP constructs at early time points (Figure 3C). However, the biggest difference was found in cells treated with monothiol-T30-NPs as the average lifetime rapidly increased, indicating fluorophore dissociation and degradation of the DNA-AuNP construct (Figure 3C,E). Kinetic analysis revealed no significant differences between PS groups (dithiol-psT30-NPs *t*_1/2_ = 63.4 h^–1^, monothiol-psT30-NPs *t*_1/2_ = 47.6 h^–1^) while the monothiol-T30-NPs were the least stable (*t*_1/2_ = 3.7 h^–1^) (Figures 3E and S4B).

**Figure 3:**
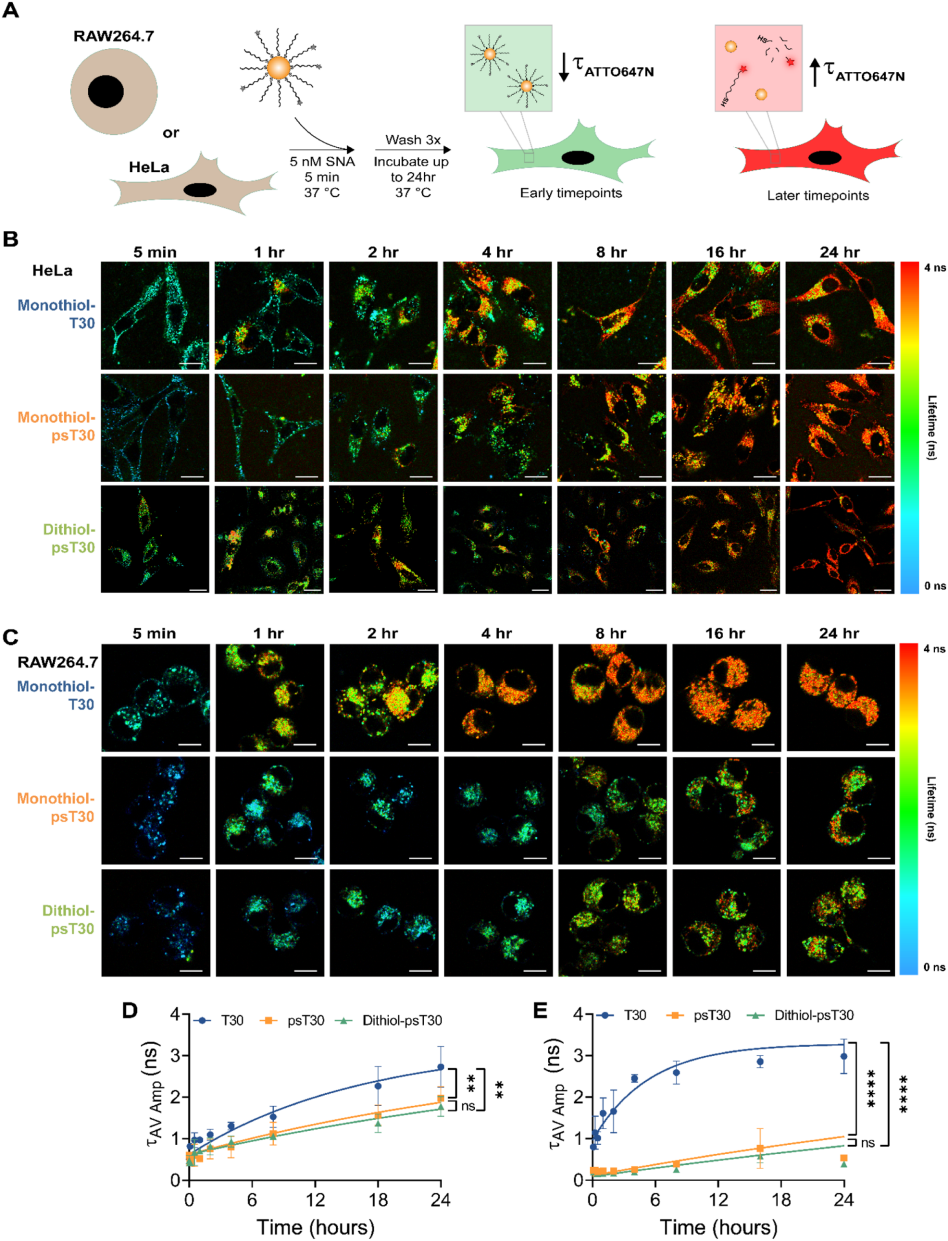
Visualization of SNA intracellular trafficking and dissociation through FLIM. **A.** Schematic showing experimental design for FLIM timelapse experiments. HeLa or RAW264.7 cells were plated overnight prior to the experiment. A rapid pulse of 5 nM SNAs (monothiol-T30, monothiol-psT30, or dithiol-psT30 DNA) was introduced to cells and imaged over a 24 h time frame using FLIM. **B.** Lifetime confocal microscopy images of SNA distribution in live HeLa cells over 24 h for all three SNA constructs (5 min, 1, 2, 4, 8, 16, and 24 h). Blue-green colors represent short lifetime values, and red-yellow colors represent long lifetime values for ATTO647N-labeled DNA. Scale bar represents 20 μm. **C.** Lifetime confocal microscopy images of SNA distribution in live RAW264.7 macrophages over 24 h for all three SNA constructs (5 min, 1, 2, 4, 8, 16, and 24 h). Blue-green colors represent short lifetime values, and red-yellow colors represent long lifetime values for ATTO647N-labeled DNA. Scale bar represents 20 μm. **D, E.** Plot showing the average amplitude-weighted lifetimes for monothiol-T30, monothiol-psT30, and dithiol-psT30 SNAs in HeLa cells (**D**) and RAW264.7 cells (**E**). Lifetimes were quantified by using a biexponential reconvolution model. Data were fit using a one-phase association model with the plateau constrained to the free ATTO647N-DNA lifetime in solution (τ_av_ amp = 3.29 n.s.). Repeated measures one-way ANOVA tests were conducted to determine significance with posthoc Tukey’s tests upon significance. *p* values are reported as ns (*p* > 0.05), ** (*p* < 0.01), and **** (*p* < 0.0001). All measurements were conducted in at least biological triplicates with multiple cells collected for each data point as technical replicates. Cells were grown in 5% CO_2_, 100% humidity, and 37 °C.

Interestingly, the calculated half-lives were much longer for PS NPs in RAW264.7 cells compared to HeLa cells, while the PO NPs showed the opposite trend of being degraded more rapidly instead. This potentially indicates higher glutathione expression within HeLa cells, which is true in many cancers compared to healthy tissue.^64,65^ To quantify the magnitude of SNA degradation in cells, we next applied lifetime:%unbound titration (Figure 1E) to the intracellular FLIM measurements (Figure S9A). This analysis provides insight into the loss of SNA function by measuring the fraction of DNA-fluorophore that is released from the AuNP rather than direct hydrolysis of the nucleic acid. Interestingly, we found that, on average, DNA-AuNP constructs show partial degradation with monothiol-T30-NPs, monothiol-psT30-NPs, and dithiol-psT30-NPs only reaching 19.5, 7.0, and 5.7% DNA degradation after 24 h of incubation (Figure S9B). However, we found that the rate of degradation was slower during the first 18 h of incubation, while the last 6 h of incubation had faster degradation, most prominent in the PO-SNA construct (Figure S9B). Applying the same analysis toward RAW264.7 macrophages, we also found that the PO-SNA construct degraded much more rapidly during the initial 6 h, with 19.7% DNA degradation over 24 h (Figure S9C). Importantly, a 20% loss of DNA density on DNA-AuNP conjugates is reported to lead to particle aggregation, and accordingly, our results suggest that the PO backbone (conventional) DNA-AuNP are likely degraded at 24 h after cell internalization.

As we narrowed down the importance of nucleases in dictating DNA-AuNP fate, we used siRNA to knockdown DNase II within HeLa cells (Figure 4A). We believed that downregulation of DNase II would lead to longer intracellular half-lives, at least for the PO NPs. Through RT-qPCR, we confirmed that the siRNA transfection was effective and significantly decreased mRNA expression at multiple concentrations after a 24 h incubation (Figures 4B and S5A-C). We further show functional protein knockdown by incubating HeLa cells with 100 nM siRNA for 48 h and running cell lysate through an SDS-PAGE gel, and subsequently performing a fluorescent Western blot (Figures 4C and S5D). After knockdown confirmation, we continued with the same treatment regime as used during the Western blot for FLIM experiments. After 48 h siRNA incubation, HeLa cells were pulsed with 5 nM DNA-AuNPs as before, washed, and imaged using FLIM over a 24 h period (Figure 4D). Qualitatively, all three DNA-AuNP constructs were visually similar within each time point, starting at short lifetimes and increasing over time. Importantly, the lifetime increase was less dramatic compared to wild-type HeLa cells, only appearing yellow/orange at the 24 h time point (Figure 4D). After biexponential reconvolution fitting, we found that DNase II knockdown significantly lengthened the intracellular half-life for the monothiol-T30-NP group (*t*_1/2_ = 27.8 h^–1^) (Figures 4E and S5E). However, DNase II knockdown did not significantly stabilize either of the PS groups despite marginally lengthening the half-lives (dithiol-psT30-NPs *t*_1/2_ = 37.4 h^–1^, monothiol-psT30-NPs *t*_1/2_ = 28.2 h^–1^) (Figures 4F,G and S5E). This suggests that DNase II is not solely responsible for DNA-AuNP degradation in cells, especially with nuclease-resistant backbone modifications.

**Figure 4:**
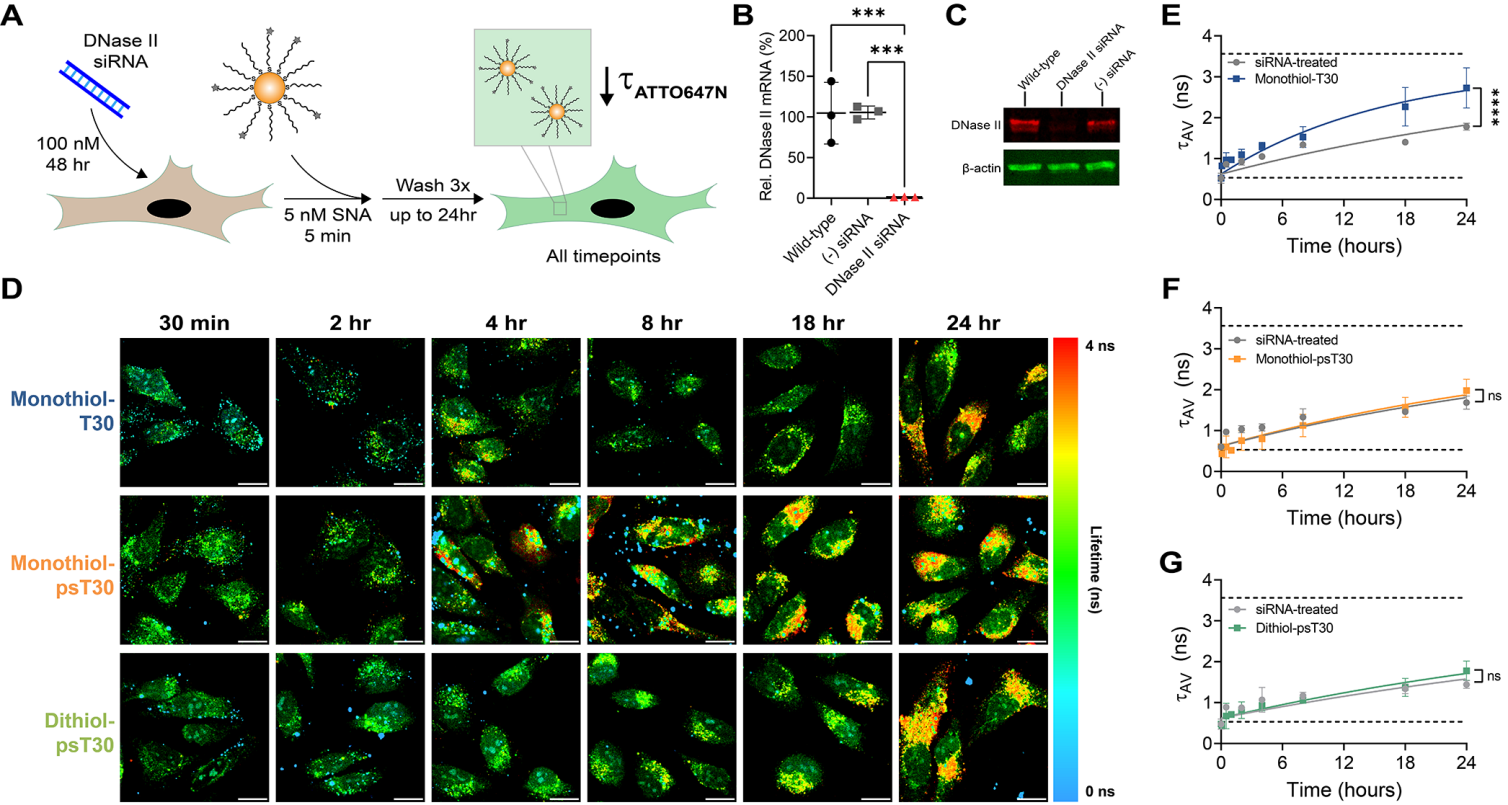
siRNA-mediated knockdown of DNase II improves the SNA circulation time. **A.** Schematic of the DNase II knockdown experimental design. HeLa cells are plated overnight and are transfected with 100 nM DNase II siRNA for 48 h. Next, cells are pulse treated with 5 nM SNA (monothiol-T30, monothiol-psT30, or dithiol-psT30) for 5 min, washed, and then imaged over a 24 h time frame using FLIM. **B.** Quantification of DNase II mRNA knockdown using RT-qPCR for wild-type HeLa cells (nontreated), 100 nM negative control siRNA, and 100 nM DNase II siRNA transfected using oligofectamine for 24 h. Relative expression is determined through the ΔΔCt method with the nontreated wild-type group as 100% expression and HPRT-1 as the housekeeping gene. **C.** Fluorescent Western blot images for wild-type HeLa cells, DNase II siRNA treated cells (100 nM), and negative control siRNA treated cells (100 nM) after 48 h. Primary anti-DNase II and anti-β-actin antibodies are labeled with Alexa Fluor Plus 647 and Alexa Fluor Plus 488 secondary antibodies, respectively. **D.** Representative lifetime confocal microscopy images for all three SNA constructs in live HeLa cells across 24 h incubation after treatment with 100 nM DNase II siRNA for 48h. Blue-green colors represent short lifetime values, and red-yellow colors represent long lifetime values for ATTO647N-labeled DNA. **E–G.** Plots showing the average amplitude lifetimes for DNase II siRNA-treated and wild-type HeLa cells after incubation with monothiol-T30 (**E**), monothiol-psT30 (**F**), and dithiol-psT30 (**G**) SNAs. Dashed horizontal lines indicate the lifetime values for 100% unbound and 100% bound nucleic acids, as derived from Figure 1. Data were fit using a one-phase association model. Statistics were conducted using an extra sum-of-squares *F* test with *p* values reported as ns (*p* > 0.05) and **** (*p* < 0.0001). All measurements were conducted in at least biological triplicates with multiple cells collected for each data point as technical replicates. Cells were grown in 5% CO_2_, 100% humidity, and 37 °C. Scale bar represents 20 μm.

Finally, we sought to use FLIM to assign discrete lifetime values that correspond to distinct endosomal maturation periods, namely, early endosomes, late endosomes, and lysosomes (Figure 5A). Using a two-pulsed-laser confocal-FLIM system, we captured two-color images with one detector channel corresponding to ATTO647N-DNA-AuNP signal as done earlier with the other detector corresponding to Alexa Fluor Plus 488, which stained for endosome markers. We then used the endosomal stain signal to create an image mask and applied it to the ATTO647N signal, where only overlapping pixels were used for FLIM fitting and analysis (Figure 5B). We chose EEA1, Rab7, and LAMP1 to stain for early endosomes, late endosomes, and lysosomes, respectively, as these markers are well-established and have high specificity for endosomal maturation.^62,66,67^ To perform this, we pulse-treated HeLa cells with all three DNA-AuNP constructs and incubated cells for up to 24 h, as before. After incubation, cells were fixed, permeabilized, and stained using primary and secondary antibodies. Visually, we can see that the FLIM images remain unaffected by the fixation process as cells show short lifetimes at early time points and long lifetimes at the 24 h time point (Figure 5C–E). Additionally, the endosomal stains do not show nuclear localization and appear visually similar across time points (Figure 5C–E). Applying the masking strategy, we found that the timelapse corresponding to each stain remains flat over the 24 h period yet differs by lifetime baseline (Figures 5F–H and S6A-C). No significant differences were found in studying the masked lifetimes across each DNA-AuNP construct, indicating that the masked lifetimes were independent of SNA dissociation (Figures 5F–H and S6A-C). To further represent the endosomal lifetimes measured, we created a “super plot”, where all time points are grouped by endosomal marker and colored by DNA-AuNP construct (Figures 5I and S6D). Here, we see that there are no trends across each construct and that distinct lifetimes values are assignable to each marker, where τ_av_^EEA1^ = 0.62 ± 0.12 ns, τ_av_^Rab7^ = 1.32 ± 0.09 ns, τ_av_^LAMP1^ = 2.33 ± 0.14 ns (Figure 5I). We next used these endolysosomal lifetime values to establish lifetime gates and determine if endosomal distribution could be measured independently of endosomal stains. We found that analysis of the DNA-AuNP lifetime distributions in HeLa cells revealed that SNAs were primarily compartmentalized by early endosomes for 1 h, transitioned to late endosomes up to 4 h, and remaining in lysosomes for the remainder of 24 h of incubation (Figure S10A-D). Interestingly, small populations of SNAs were found to reside within early endosomes or late endosomes for the entire 24 h duration, which suggests that endosomal trafficking may be hindered by sequestered SNAs (Figure S10D,E). Lastly, as the fluorescence lifetime is total intensity-independent/concentration-independent, we measured the ratio of masked fluorescence intensity to total cell intensity to confirm that the DNA-AuNPs were progressing throughout the endosomal maturation process and could be evaluated through intensity-based approaches as validation. Indeed, we found that the peak ratios differed for each endosomal marker as the maximum masked overlap for EEA1, Rab7, and LAMP1 occurred at 4, 18, and 24 h, respectively (Figures 5J–L and S10E).

**Figure 5:**
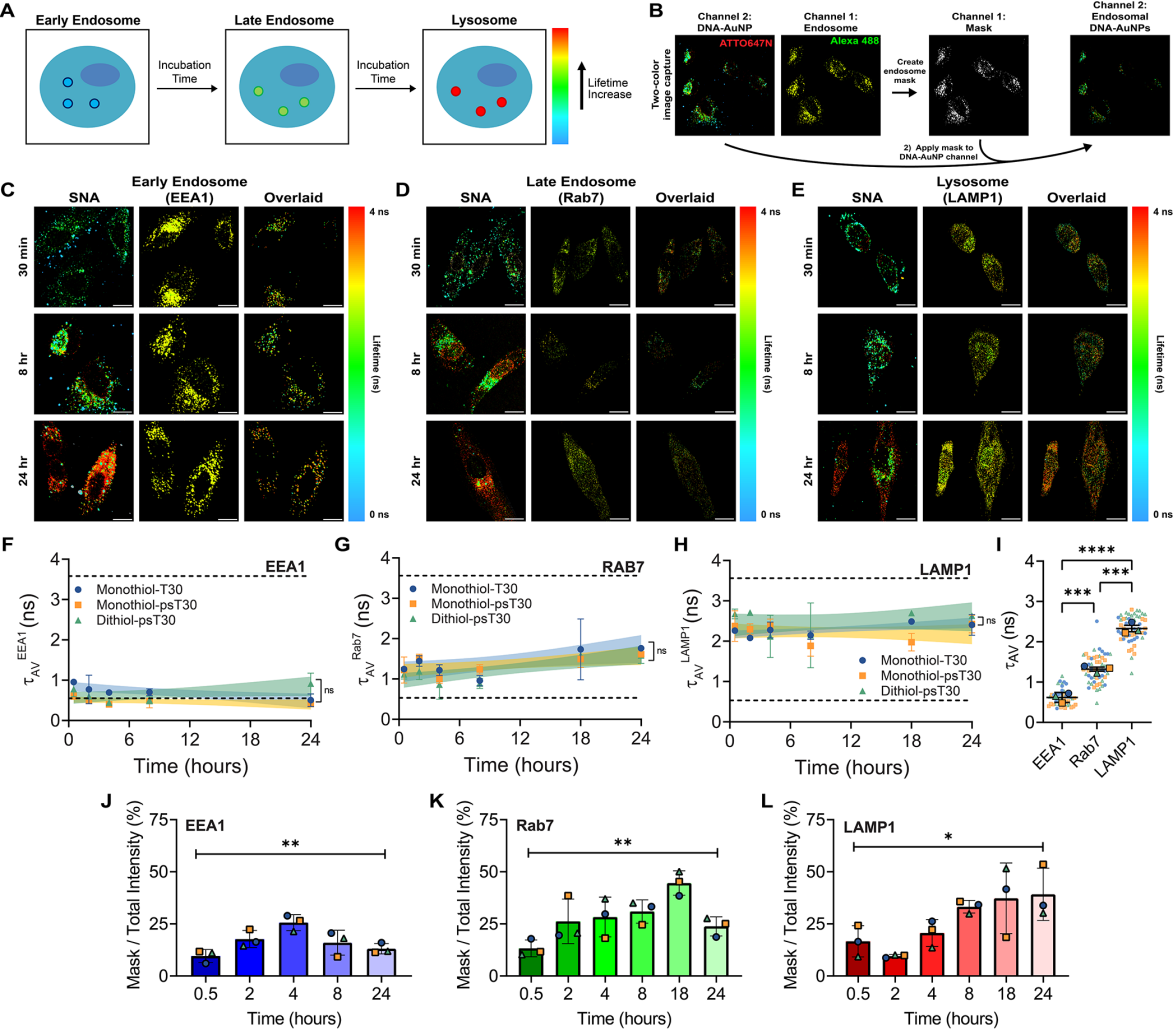
Endosomal compartmentalization reveals distinct lifetimes with two-color FLIM measurements. **A.** Schematic representing the lifetime shift with endosomal maturation from early endosomes (blue, short lifetimes) to late endosomes (green, middle lifetimes) to lysosomes (red, long lifetimes). **B.** Schematic showing the image collection process. Images are captured using two lasers (640 and 485 nm) that are separated by a dichroic mirror into two detector channels. Data are analyzed by thresholding pixels from channel 1 (Alexa Fluor Plus 488) to create a region of interest (ROI) that only contains endosomal signal. This ROI is applied to channel 2 (ATTO647N-SNA) signal and quantified using a biexponential reconvolution fitting model to determine the average amplitude lifetime. **C–E.** Representative lifetime confocal microscopy images for early endosomes (EEA1, **C**), late endosomes (Rab7, **D**), and lysosomes (LAMP1, **E**) in HeLa cells after a 5 min pulse of 5 nM SNA (monothiol-T30 shown) with 30 min, 8 h, and 24 h incubations. SNA signal (channel 2: ATTO647N), endosomal stain (channel 1: Alexa Fluor Plus 488), and overlaid signal (ROI from channel 1 onto channel 2) images are shown from left to right. Blue-green colors represent short lifetime values, and red-yellow colors represent long lifetime values for ATTO647N-labeled DNA. Note: these experiments require fixation and permeabilization to label endosomes in HeLa cells unlike previous figures. Scale bars represent 20 μm. **F–H.** Plots showing the average amplitude lifetimes of all three SNA constructs (monothiol-T30 in blue, monothiol-psT30 in yellow, and dithiol-psT30 in green) for EEA1 (**F**), Rab7 (**G**), and LAMP1 (**H**) staining over a 24 h time frame. Dashed horizontal lines indicate the lifetime values for 100% unbound and 100% bound nucleic acids as derived from Figure 1. Data are fit using a linear regression with the 95% confidence interval shown for each SNA construct. The number of cells analyzed at each time point in **F**, **G**, and **H** were *n* > 10 cells for each replicate. **I.** Super plot showing the average amplitude lifetimes for EEA1, Rab7, and LAMP1 staining in HeLa cells with all time points grouped together. Monothiol-T30 (blue circles), monothiol-psT30 (yellow squares), and dithiol-psT30 (green triangle) are shown with the large shapes representing the mean across the 24 h timelapse and smaller shapes representing each individual data point. **J–L**. Plots showing the percentage of masked fluorescence intensity over total fluorescence intensity were obtained using endosomal stains EEA1 (**J**), Rab7 (**K**), and LAMP1 (**L**). Note: the same masks for **F–H** were used for **J–L**. For **F–I**, statistics were conducted using a repeated measures one-way ANOVA with posthoc Tukey’s tests upon significance. *p* values are reported as ns (*p* > 0.05), *** (*p* < 0.001), and **** (*p* < 0.0001). The number of cells analyzed at each time point in **J**, **K**, and **L** were *n* > 10 cells for each replicate. For **I**, statistical comparisons were conducted using each SNA mean (*n* = 3) rather than individual data points (*n* > 40). Cells were grown in 5% CO_2_, 100% humidity, and 37 °C. All experiments were conducted in biological triplicate.

## Conclusion

In this work, we showcase the benefits of FLIM in monitoring the integrity of SNAs, specifically DNA-AuNPs. Fluorescence lifetime is unique, as it is an intrinsic property of a fluorophore, which is independent of concentration and instead dependent on its local microenvironment. Traditionally, DNA-AuNP integrity has been measured via colocalization of the NP core and DNA via correlative imaging and ratiometric measurements of multiple fluorophores on AuNPs;^49,68^ however, these measurements are indirect and have the potential to skew results as DNA-AuNPs are entrapped within endosomes and are trafficked through the cell. Here, we establish that FLIM can detect AuNP quenching of ATTO647N via NSET to result in binary lifetime states. Because of this large lifetime shift, we were led to perform titration measurements and found that FLIM is sensitive to detect a subtle loss of nucleic acid from the SNA.

To study the parameters governing the intracellular fate of DNA-AuNPs, our three designed DNA-AuNP constructs exhibit varying degrees of stability against nucleases and thiol-displacement through DNA backbone modifications and thiol modifications. Importantly, the benefit of PS modifications to enhance DNA-AuNPs nuclease stability is not well-established in the literature.^69^ Less commonly addressed is the role of endogenous thiol-displacing agents (i.e., glutathione) as covalent linkages are not susceptible to this effect.^30^ We show that the incorporation of a dithiol group increases DNA-AuNP resistance to a thiol-displacing agent, DTT, over monothiol groups. Additionally, the incorporation of PS backbone modifications allows the DNA-AuNP to become highly resistant to nucleases such as DNase I and II. After exposing cells to our three constructs, we found that PO DNA-AuNPs are more greatly degraded, while PS DNA-AuNPs are more stable, implicating nucleases as driving factors of the intracellular fate. Cell-type is important, as DNA-AuNP degradation in HeLa cells is less distinct across all three constructs, while there is a large difference between PO and PS constructs with RAW264.7 macrophage stability. This suggests lower nuclease expression and higher thiol-displacement in HeLa cells, while macrophage degradation is mainly driven by nuclease activity.

Interestingly, we found that monothiol-T30, monothiol-psT30, and dithiol-psT30 SNA constructs degraded at 19.5, 7.0, and 5.7% after 24 h post incubation. While this loss of DNA seems modest, this is substantial as 20% loss of DNA density leads to aggregation in conventional DNA-AuNP conjugates and hence suggests that the SNAs are likely aggregated for conventional PO modified DNA. When we downregulated DNase II through RNA interference (RNAi), the PO construct became significantly more stable, while the PS constructs showed no improvement. Excitingly, this result highlights DNase II’s involvement in PO DNA-AuNP degradation; however, PS DNA-AuNPs dissociation is not driven by DNase II and instead by alternative mechanisms such as thiol-displacement. We conclude this work by showing that FLIM can be used to assign lifetime values corresponding to endosomal maturation markers. As the progression from early endosomes to late endosomes, and finally lysosomes is well-known, we found that our masking strategy using known endosome markers provided time-independent lifetimes. This establishes that not only does fluorescence lifetime reveal the composition of DNA-AuNPs, but lifetime can also be correlated directly with endosomal maturation. Overall, we believe that incorporating DNA backbone modifications is a valuable strategy to improve SNA stability, as DNase II actively degrades PO DNA constructs. We also believe that FLIM is a powerful tool to characterize intracellular delivery for nanoparticles and that this work demonstrates the unique potential of this tool for biological applications.

## Methods

### Materials

All chemicals purchased were used without further purification, unless otherwise noted. Sodium bicarbonate (Cat# S6014-500g), sodium acetate (Cat# S2889-250G), MES (Cat# M8250-100G), sodium citrate tribasic dihydrate (Cat# S4641-500G), hydrochloric acid (Cat# HX0603-3), DMSO (Cat# MX1457-7), acetonitrile (Cat# 34998-4L), potassium cyanide (Cat# 60178-25G), sodium chloride (Cat# SX0420-1), sodium dodecyl sulfate (Cat# L3771-100G), ammonium persulfate (Cat# A3678-100G), TEMED (Cat# T9281-50 ML), DNase II (Cat# D4138-20KU), Bovine serum albumin (Cat# 10735078001), DTT (Cat# 10197777001), and formaldehyde solution (Cat# 47608-250 ML-F) were purchased from Sigma-Aldrich. Tetrachloroauric (III) acid trihydrate (Cat# 411070010) was purchased from Acros Organics. HEPES (Cat# 5380) and glycine (Cat# 4810) were purchased from OmniPur. Quant-IT Oligreen ssDNA reagent (Cat# 22360), oligofectamine (Cat# 58303), and nitrocellulose membranes (Cat# LC2001) were purchased from Invitrogen. Opti-MEM I Reduced Serum Medium (Cat# 31985070), Dulbecco’s Modified Eagle Medium 1× (Cat# 11995-065), and 0.4% Trypan Blue (Cat# 15250-061) were purchased from Gibco. Nitric acid (Cat# T003090500), glutaraldehyde (Cat# A17876.AP), PageRuler Plus Prestained Protein Ladder (Cat# 26619), negative control siRNA (Cat# AM4611), Pierce RIPA buffer (Cat# 89900), DNase I (Cat# EN0521), Micro BCA Protein Assay Kit (Cat# 23235), primary antibodies: beta actin (Cat# MA515739), DNase II (Cat# PA519900), EEA1 (Cat# MA514794), Rab7 (Cat# PA523138), LAMP1 (Cat# MA1164), and secondary antibodies (Cat# A32731TR, A32733TR, A32766TR) were purchased from ThermoFisher. 10× tris/glycine/SDS buffer (Cat# 1610732), 10× TBS (Cat# 1706435), 4× Laemmli buffer SDS (Cat# 1610747), Trans-Blot Turbo 5X Transfer Buffer (Cat# 10026938), 30% acrylamide/bis solution 29:1 (Cat# 1610156), 20× PBS Tween-20 (Cat# 28352), and Bio-Gel *p*-2 Gel (Cat# 1504118) were purchased from Bio-Rad. Triton-X-100 (Cat# BP151-100) and Tween 20 (Cat# BP337-500) were purchased from Fisher Scientific. 0.25% trypsin, 2.21 mM EDTA, 1× sodium bicarbonate (Cat# 25-053-Cl), and fetal bovine serum (Cat# 35-010-CV) were purchased from Corning. ATTO647N-NHS ester (Cat# 2856) was purchased from AAT Bioquest. SMARTPool ON-TARGETplus Human DNASE2 siRNA (Cat# L-009667-00-0005) was purchased from Horizon. Triethylammonium acetate (Cat# 60-4110-62) was purchased from Glen Research. QIAzol lysis reagent (Cat# 79306) and RNeasy Mini Kit (Cat# 74106) were purchased from QIAGEN. DNase I Set (Cat# E1010) was purchased from Zymo Research. High-Capacity cDNA Reverse Transcription Kit (Cat# 4368814) was purchased from Applied Biosystems. Penicillin/streptomycin (Cat# K952-100 mL) and PerfeCTa SYBR Green FastMix ROX (Cat# 95073-012) were purchased from VWR. All oligonucleotides were purchased from Integrated DNA Technologies, stored at −30 °C and used without further purification. Oligonucleotides were modeled using NUPACK software and Integrated DNA Technologies, OligoAnalyzer, tool. Nanopure water (Barnstead Nanopure system, resistivity = 18.2 MΩ) was used to prepare stock solutions. UB4 buffer was prepared by adding 20 mM sodium acetate, 20 mM MES, 20 mM HEPES, and 117 mM NaCl.

### Cell Culture

HeLa cells and RAW264.7 macrophages were obtained from ATCC and cultured according to the ATCC guidelines. HeLa cells were cultured in DMEM with 10% FBS, penicillin (100 U/mL), and streptomycin (100 mg/mL). RAW264.7 cells were cultured in DMEM with 10% FBS, penicillin (100 U/mL), streptomycin (100 mg/mL), sodium bicarbonate (1500 mg/L), sodium pyruvate (1 mM), and l-glutamine (2 mM). Opti-MEM I Reduced Serum Medium was used for siRNA transfection. Cells were incubated with 100% humidity and 5% CO_2_ at 37 °C. Cells were passaged at ∼70–80% confluency following ATCC guidelines. Experiments were conducted only on cells under passage 15. Cells were counted using a hemocytometer with trypan blue on an Echo Rebel microscope.

### Synthesis of Dye-Functionalized DNA

Amine-modified DNA was functionalized to the ATTO647N NHS ester via NHS ester amine chemistry. A 50-μg aliquot of ATTO647N NHS ester was suspended in 30 μL of fresh DMSO. ATTO647N solution was combined with a solution containing 40 μL H_2_O, 10 μL volume of 10× PBS, 10 μL of 1 M NaHCO_3_, and 10 μL of 1 mM DNA. The reaction was left for 1 h. Afterward, the reaction mixture was run through a 0.2 μm pore spin-column with a *p*-2 gel (Bio-Rad) to remove excess unreacted dye. Product was purified through reverse-phase HPLC with an Agilent AdvanceBio Oligonucleotide C18 column and eluted in solvents A: 0.1 M TEAA in H_2_O and B: acetonitrile (ACN). Product was eluted with a linear gradient of 10–27.5% solvent B over 35 min. Product was concentrated using a VacuFuge and confirmed using electron spray ionization mass spectrometry (Figure S11 and Table S3). UV–vis spectroscopy (Nanodrop 2000c) was also used to confirm the product by ensuring that the ATTO647N:DNA ratio was 1:1 (A647:A260).

### Synthesis of 15 nm Gold Nanoparticles

Spherical gold nanoparticles (AuNPs) with a diameter of 15 nm were prepared as previously described^36^ and following the Turkevich–Frens method.^70,71^ A 250 mL two-neck round-bottom flask and a stir bar were cleaned by adding aqua regia (3:1 HCl to HNO_3_) and mixing for 1 min. Note: Aqua regia is corrosive, and care must be taken to ensure safety when handling and disposing of the solution. Aqua regia was discarded into a designated waste container and rinsed with nanopure water (∼15 times). The flask was inverted and left to dry overnight. Before the synthesis, the flask and stir bar were suspended in a water bath. A condenser was attached to one neck, and the other neck was covered with foil. First, 98.9 mL of H_2_O was added to the flask along with 0.1 mL of 0.25 M HAuCl_4_ solution in water. The solution was boiled, and a 1 mL solution of sodium citrate tribasic (30 mg/mL) was swiftly injected into the flask and refluxed for 10 min. The flask was rapidly placed on ice to cool and was stored at 4 °C until further use. The concentration was determined through UV–vis spectroscopy by measuring the peak absorbance (∼520 nm) and using Beer–Lambert’s Law: *A* = ε × *c* × *l*, where ε = 3.1 × 10^8^ cm^–1^ M^–1^, *l =* 0.1 cm. Note: the extinction coefficient is dependent on AuNP size as determined by TEM.

### DNA Functionalization of Gold Nanoparticles

AuNPs were functionalized as described previously^36^ and following the freeze method.^24,72^ Briefly, thiolated DNA (monothiol-T30, monothiol-psT30, and dithiol-psT30) was added in 300-fold molar excess to 15 nm AuNPs. To avoid homo-FRET interactions, a 1:10 ratio of ATTO647N-DNA to unlabeled DNA was used across all experiments. The DNA-AuNP was frozen at −30 °C for 1 h or at −80 °C for 10 min. Next, the SNA solution was thawed at RT for ∼30 min. The solution was brought up to 500 μL in H_2_O or split into 500 μL aliquots to ensure AuNP sedimentation during centrifugation. The solution was then washed three times by centrifugation at 13,000 × *g* for 20 min at RT (Eppendorf Centrifuge 5424R), carefully aspirating the supernatant and resuspending the solution in 500 μL H_2_O. The SNA solution was left concentrated (∼100 μL) after the final wash and characterized through TEM, DLS, and UV–vis spectroscopy by measuring the peak absorbance (∼527 nm) and using Beer–Lambert’s Law (Figure S12).

### Quantification of Nucleic Acid Density

Spherical nucleic acids were prepared as described above. A five-point standard curve was prepared with 100 μL of 2, 20, 50, 100, and 200 nM ATTO647N-labeled DNA in 1× TE buffer. SNA samples were prepared as 100 μL of 0.5 nM AuNP in 1× TE buffer. A 5 μL volume of 1 M KCN solution was added to each sample and incubated for 30 min at RT to etch the AuNP core. Note: KCN in aqueous solution must be handled in a fume hood and precautions must be taken with buffer conditions to maintain safety. Next, 105 μL of 1× OliGreen ssDNA reagent in 1× TE buffer was added to each sample and fluorescently measured via a plate-reader spectrophotometer (BioTek Synergy H1 Hybrid Multi-Mode Reader). OliGreen measurements used a 510 nm long-pass dichroic mirror with bandpass excitation and emission filters at 485/20 and 528/20 nm, respectively. ATTO647N measurements used a 660 nm long-pass dichroic mirror with bandpass excitation and emission filters at 620/40 and 680/30 nm, respectively. Standards were fit to a linear regression, and sample concentration was determined by interpolation. Sample concentration from OliGreen was divided by the initial AuNP concentration to determine # total DNA/AuNP. ATTO647N concentration was used to determine # ATTO647N-DNA/AuNP.

### Confocal-FLIM Microscope Setup

All images were acquired on a Nikon Ti2 Eclipse inverted confocal microscope equipped with a Picoquant Laser Scanning TCSPC upgrade. Samples were prepared on black 96-well glass-bottom optical plates (Nunc) and mounted to the microscope using a well-plate stage attachment. A Plan Apo Lambda 60×/1.40 oil objective was used for cell samples, and a Plan Apo VC 20×/0.75 air objective was used for solution measurements. All images were collected as multiframe 512 × 512-pixel images for 2 min with a 1.9 μs dwell time and 40 μm pinhole (∼1 AU) using a PMA hybrid 40 dual detector. For most measurements, samples were pulsed with a 640 ± 10 nm laser at 20 MHz and signal was collected on one channel (channel 2) after passing a long-pass dichroic mirror and 690/45 nm bandpass emission filter. With two-color measurements (endosomal staining experiments), samples were pulsed with a 640 ± 10 nm laser and a 485 ± 10 nm laser at 20 MHz. Signal was separated through a 640 nm long pass dichroic mirror with reflected light (shorter, Alexa Fluor Plus 488) passing a 520/47 nm bandpass filter into channel 1, and transmitted light (longer, ATTO647N) passing a 690/45 nm bandpass filter into channel 2. Realtime signal was attenuated to reduce the photon pile-up effect (signal <1% laser pulse rate). The instrument response function (IRF) was measured using a saturated and quenched Coomassie Blue solution in KI after imaging sessions. Data were processed using SymPhoTime64 (PicoQuant) software. ROIs were drawn around cell regions in combination with a 25-photon count pixel threshold. Lifetime decay profiles were quantified using a biexponential reconvolution fitting algorithm with the average amplitude lifetime value chosen for primary statistical analysis.

### RT-qPCR to Assess DNase II Levels after siRNA Treatment

Prior to transfection, 5 × 10^4^ HeLa cells were seeded on a culture-treated 12-well plate in DMEM. T25 μM, 10 μM, and 1 μM stocks of negative control siRNA and DNase II siRNA were prepared as well. The following morning, cells were washed once with warm HBSS and left in 400 μL of Opti-MEM for 20 min. During this period and following the oligofectamine (OFA) manufacturer’s procedure, 5 μL of siRNA stocks were diluted in 85 μL of Opti-MEM, while 2 μL of OFA was diluted in 8 μL of Opti-MEM in separate tubes. After 5 min, diluted siRNA and diluted OFA were combined to form complexes. After 15 min, 100 μL of the siRNA-OFA complex solution was added to cells. For wild-type/nontreated cells, a solution containing 100 μL of Opti-MEM was added instead. After 4 h incubation, an additional 250 μL of 30% FBS in DMEM without antibiotics were added to each well to create a final concentration of 10% FBS for the remainder of the 24 h incubation. After the incubation, cells were washed with HBSS and lysed using 350 μL of QIAZOL lysis reagent and 350 μL of 70% ethanol. Total RNA was collected following the procedure described by the QIAGEN RNeasy Mini Extraction Kit . RNA samples with a poor A260/A280 ratio or <20 ng/μL concentration were discarded. RNA was reverse transcribed following the High-Capacity cDNA Reverse Transcription Kit using a Bio-Rad T100 Thermal Cycler. DNase II mRNA levels were quantified following the PerfeCTa SYBR Green FastMix RT-qPCR two-step protocol with 500 nM primers (Table S2) in a Roche LightCycler 96. Relative mRNA quantification was performed by using the ΔΔ*C*_t_ method with HPRT-1 as an internal control with technical replicates for each sample.

### Western Blot to Confirm DNase II Protein Knockdown

HeLa cells were transfected with siRNA in a manner similar to that of RT-qPCR experiments with subtle differences. Instead, 1.5 × 10 ^5^ HeLa cells were plated on culture-treated 6-well plates and siRNA treatment was scaled according to the Oligofectamine manual. Cells were incubated for 48 h as well. After incubation, cells were washed once with HBSS before 150 μL of chilled RIPA lysis buffer was added to each well. The plate was placed on ice for 15 min on a shaker. Next, a cell scraper was used to collect lysates, which were then pipetted into separate tubes and placed on ice. Tubes were centrifuged at 14,000 × *g* for 15 min at 4 °C and the supernatants were collected for each sample (∼100 μL). A micro BCA protein assay kit was used to quantify total protein concentration following the manufacturer’s protocol with nine standards of known BSA protein concentration. Both 1:20 and 1:40 dilutions were prepared for each sample. All standards and samples were mixed with equal volumes of BCA working reagent and incubated at 37 °C for 2 h. The absorbance at 562 nm was measured using a plate reader (BioTek Synergy H1 Hybrid Multimode Reader), and standards were fit to a linear regression. Sample concentration was interpolated from the fit, and all concentrations were normalized to 2 mg/mL.

To prepare samples for SDS-PAGE, 15 μL of protein samples were combined with 5 μL of 4× Laemmli loading buffer and further denatured for 10 min at 90 °C. An SDS-PAGE gel was cast with 4% stacking gel and 15% resolving gel components, and samples were loaded in separate wells alongside a 10–250 kDa protein ladder. The gel was run at 50 V for 45 min before increasing the voltage to 200 V for 2.5 h in 1× SDS/glycine/tris running buffer on a Bio-Rad PowerPac Basic system. The gel was transferred to a nitrocellulose membrane using a Trans-Blot Turbo transfer system and washed multiple times with 1× TBS-T solution. The blot was blocked with 3% BSA in 1× TBS-T for 1 h at RT before an overnight incubation with primary antibodies (1:1000 rabbit anti-DNase II, 1:2000 mouse β-actin) in blocking buffer at 4 °C. The following morning, blots were washed 5× with 1× TBS-T and incubated with secondary antibodies (1:10,000 goat antirabbit Alexa Fluor Plus 647 and 1:10,000 donkey antimouse Alexa Fluor Plus 488 antibodies) in blocking buffer for one h at RT. Blots were washed with 1× TBS and imaged using an iBright FL1500 Imaging System.

### FLIM Measurements of SNAs in Solution

Briefly, 1:10 monothiol-T30, 1:10 monothiol-psT30, and 1:10 dithiol-psT30 SNA constructs were prepared as above and added to a black 96-well glass-bottom optical plate (Nunc) at a concentration of 5 nM in 1× PBS. Note that each AuNP is functionalized to ∼150 DNA strands, and of those, 15 strands are labeled with ATTO647N. Free ATTO647N-DNA (50 nM) corresponding to each SNA was also prepared in 1× PBS. All measurements were conducted in 150 μL volumes at RT and imaged through confocal-FLIM as described above. For titration experiments, solutions of monothiol-T30 SNAs were prepared in multiple wells and measured at 0.5 nM concentrations in 1× PBS. Additionally, monothiol-T30-ATTO647N DNA was serially diluted and added to each well to create final concentrations of 50, 10, 5, 1, 0.5, 0.1, and 0 nM in addition to the 0.5 nM SNA. A positive control of 50 nM DNA only was prepared, as well. Data were fitted using a dose-response sigmoidal function in GraphPad Prism.

For SNA stability experiments against nucleases, all three SNA constructs were prepared in optimized buffers for each enzyme according to supplier recommendations. DNase I buffer was composed of 10 mM Tris-HCl, 2.5 mM MgCl_2_, and 0.1 mM CaCl_2_ at pH 7.5 in nanopure H_2_O. DNase II buffer was composed of 1× UB4 buffer at pH 5.0, which is 20 mM HEPES, 20 mM MES, 20 mM sodium acetate, and 117 mM NaCl in nanopure H_2_O. 5U of DNase I and II were added to 0.5 nM SNA solutions in the corresponding buffer and imaged using FLIM. Measurements were taken at various time points after RT incubation: 0 min (before enzyme addition), 15 min, 30 min, 1, 2, 4, and 24 h. For DTT stability experiments, all three SNA constructs were prepared at 0.5 nM in 1× PBS. DTT was added at a final concentration of 100 μM and FLIM measurements were taken after 0 min (before DTT), 5 min, 30 min, and 1 h incubations at RT.

### FLIM Imaging of RAW264.7 and HeLa Cells

RAW264.7 cells or HeLa cells were plated on black 96-well glass-bottom optical plates at a seeding density of 1 × 10^4^ or 5 × 10^3^ cells, respectively, and left to adhere overnight. The following day, cells were washed with DMEM media and incubated with 5 nM of monothiol-T30, monothiol-psT30, or dithiol-psT30 SNAs for 5 min. Afterward, cells were carefully washed with media and left to incubate for time periods up to 24 h for HeLa cells or 48 h for RAW264.7 cells. After incubation, live cells were washed with FluoroBrite DMEM medium and imaged. For DNase II knockdown studies in HeLa cells, cells were transfected with 100 nM DNase II siRNA for 48 h prior to SNA incubation, following the transfection protocol above.

For endosomal staining experiments, cells were fixed, permeabilized, and stained for EEA1 (early endosome), Rab7 (late endosome), or LAMP1 (lysosome). Briefly, HeLa cells were incubated with SNAs as mentioned above for 24 h. Incubation times were staggered, so all incubations concluded at the same time. After incubation, cells were washed 2× with warm HBSS before fixing with a chilled 4% formaldehyde + 1% glutaraldehyde solution in 1× PBS for 10 min at RT. Next, cells were washed and incubated with a 0.1 M glycine solution in 1× PBS to quench unreacted aldehydes for 30 min. Cells were washed 3× with 1× PBS and permeabilized with 0.2% Triton X-100 in 1× PBS for 15 min. Permeabilized cells were washed 3× with 1× PBS-T and blocked using 0.2 μM-filtered, 3% BSA solution in 1× PBS-T for 1 h at RT. After blocking, cells were washed once with 1× PBS-T and incubated overnight with diluted primary antibodies (1:100 EEA1, 1:200 Rab7, 1:100 LAMP1) in blocking buffer at 4 °C. The following morning, cells were washed 3× with 1× PBS-T and then incubated with 1:1000 diluted Alexa Fluor Plus 488-secondary antibodies in blocking buffer for 1 h at RT. Cells were washed 3× with 1× PBS and imaged using two-color FLIM.

## Supplementary Material



## Notes

Tables of oligonucleotide sequences and their mass spectrometry analysis; additional data and experiments; further detailing of analysis techniques (PDF)
