# Supplementary material for: Exploring the Subcellular Localization and Degradation of Spherical Nucleic Acids Using Fluorescence Lifetime Imaging Microscopy
Source: ACS Nano. 2025 Jun 9;19(24):21983–96. doi: 10.1021/acsnano.5c00177 (PMC12203632; doi:10.1021/acsnano.5c00177)
Supplement: Supplementary file 1 [file nn5c00177_si_001.pdf]

Exploring the subcellular localization and degradation of spherical nucleic acids using fluorescence lifetime imaging microscopy

Steven Narum[1], Jiahui Zhang[1], Binh L. N. Vo[2], Joseph Nicolas Mancuso[2], and Khalid Salaita[1,2,*]

[1]Department of Biomedical Engineering, Georgia Institute of Technology and Emory University, Atlanta, GA 30322

[2]Department of Chemistry, Emory University, Atlanta, GA 30322

[*]*To whom the correspondence should be addressed. Email: k.salaita@emory.edu*

Supporting Table 1: Representative examples of single-stranded DNA SNA applications in cells

Technology / Application	Description	Representative References
Antisense therapies	Antisense oligonucleotide conjugated to nanoparticle; mRNA disruption	1, 2
Immunomodulation	CpG-DNA conjugated to nanoparticle to activate TLRs	3, 4
Biosensors	Applications of ssDNA-gold nanoparticles motors in live cells	5, 6
Nanozymes / DNAzymes	ssDNA DNAzyme-conjugated gold nanoparticles for applications in living cells	7-9
Diagnostics	ssDNA binding to miRNA target and detected via spectroscopic shift	10
Clinical Therapeutic (AST-008)	Cavrotolimod, a Nanoparticle Toll-like Receptor 9 Agonist, Inhibits Tumor Growth and Alters Immune Cell Composition in Mouse Models of Skin Cancer	11
Clinical Therapeutic (AST-005)	Topically Delivered Tumor Necrosis Factor-α-Targeted Gene Regulation for Psoriasis	12

Supporting Table 2. Oligonucleotide sequences and structures

Name	Sequence (5’ to 3’)
**Monothiol-T30**	/5AmMC6/TTTTTTTTTTTTTTTTTTTTTTTTTTTTTT/3ThioMC3-D/
**Monothiol-psT30**	/5AmMC6/T*T*T*T*T*T*T*T*T*T*T*T*T*T*T*T*T*T*T*T*T*T*T*T*T*T*T*T*T*T*/3ThioMC3-D/
**Dithiol-psT30**	/5AmMC6/T*T*T*T*T*T*T*T*T*T*T*T*T*T*T*T*T*T*T*T*T*T*T*T*T*T*T*T*T*T*/3DTPA/
**pH-strand**	/5AmMC6/TTGCATTGCAmCmUmGmUGCTCAGGCTAGCTACAACGAGGTGTmCmUmUmU
**DNase II forward primer**	TCGCCTTCCTGCTCTACAAT
**DNase II reverse primer**	CCCATCTTCGAGAACTGAGC
**HPRT-1 forward primer**	CTCATGGACTGATTATGGACAGGAC
**HPRT-1 reverse primer**	GCAGGTCAGCAAAGAACTTATAGCC

KEY: * refers to phosphorothioate. m_ refers to O-methyl RNA bases. Structure of 3’ Dithiol IDT modification not available online. IDT modifications are shown below.

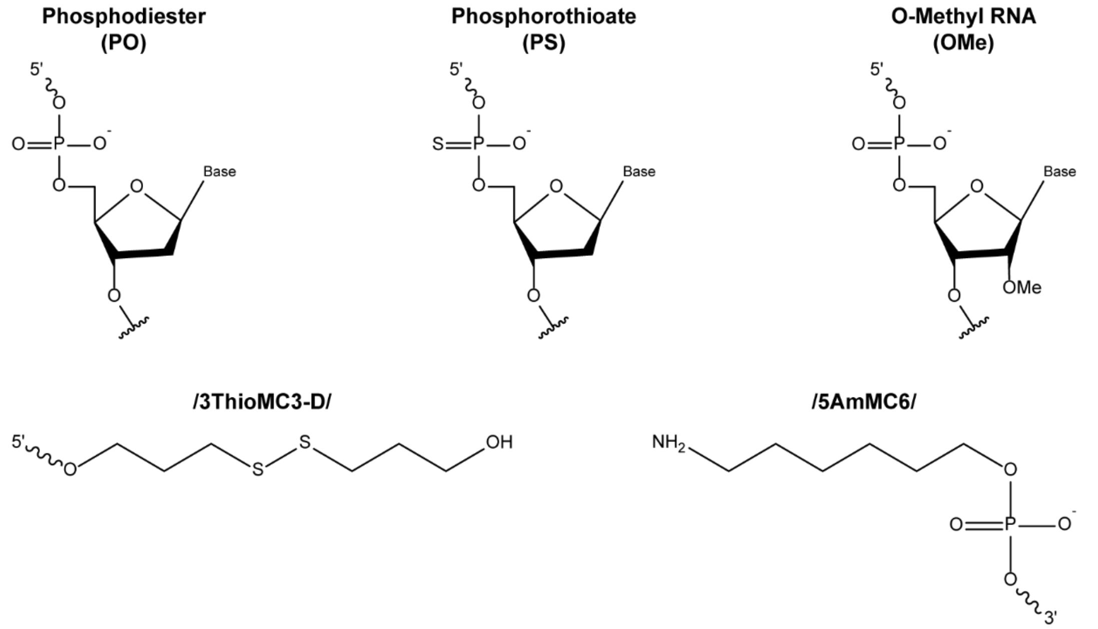

Supporting Table 3: Theoretical MW and ESI-MS experimentally measured MW for ATTO647N-oligos.

Sample	Theoretical MW (g/mol)	Measured MW (g/mol)	ΔMW (g/mol)
Monothiol-T30-ATTO647N	10115.21	10113.95	1.26
Monothiol-psT30-ATTO647N	10581.01	10580.27	0.74
Dithiol-psT30-ATTO647N	10550.91	10550.22	0.69

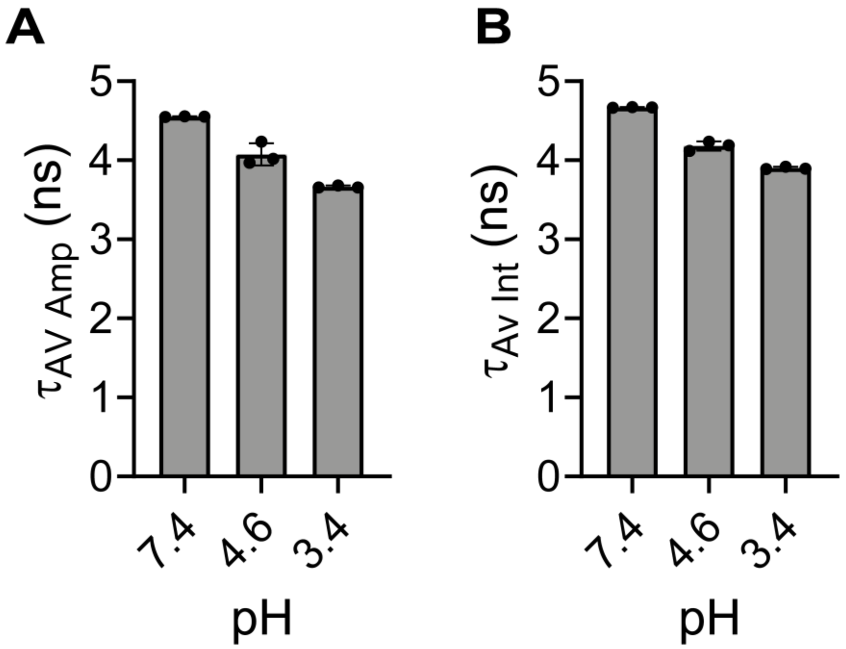

Figure S1: Measurement of ATTO647N-DNA pH-dependence using FLIM. A. Plot showing the average amplitude-weighted lifetimes of 500 nM ATTO647N-DNA (pH-strand ATTO647N) in 1X PBS buffers with pH 7.4, 4.6, or 3.4. **B.** Plot showing the average intensity-weighted lifetimes 500 nM ATTO647N-DNA in 1X PBS (157 mM Na^{+} with 0 M Mg^{2+}) buffers with varying pH. All data was fit using a bi-exponential reconvolution fitting algorithm with measurements conducted in triplicate at RT.

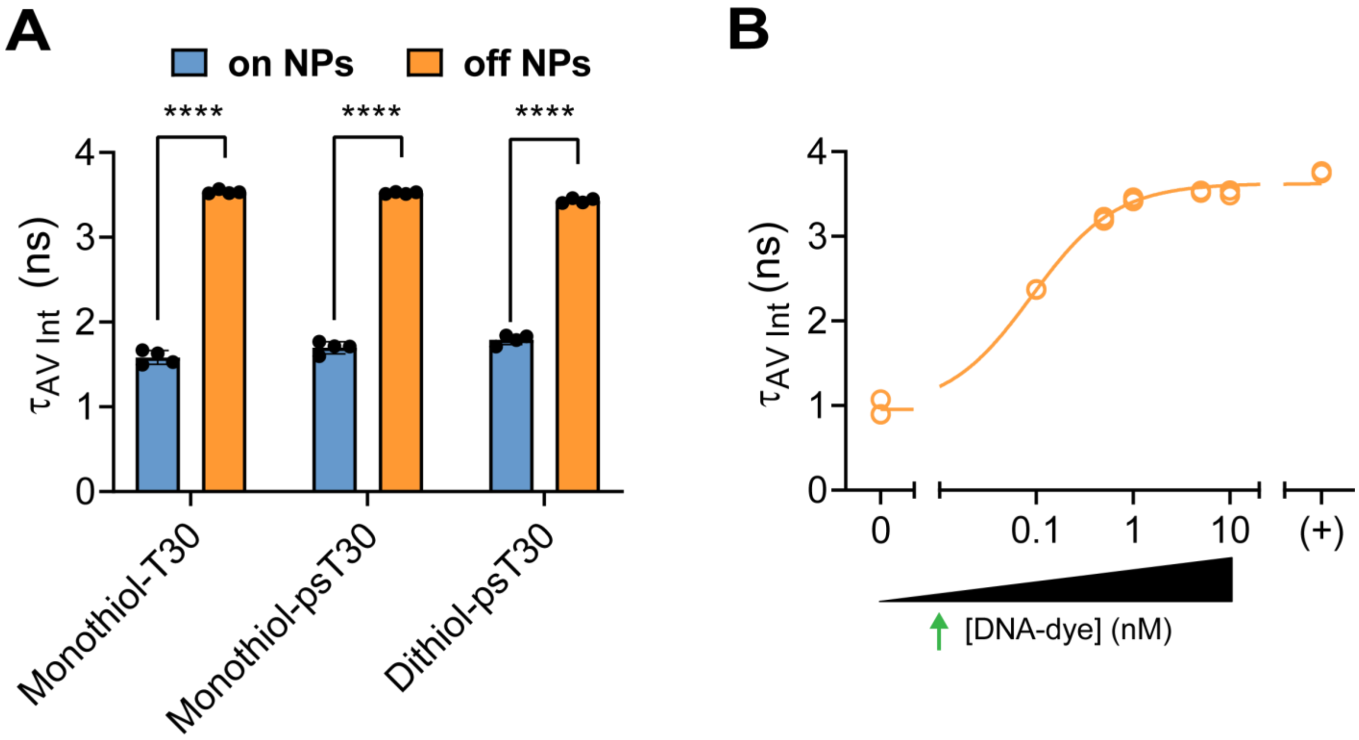

Figure S2: Intensity-weighted FLIM solution measurements of SNAs. A. Plot showing the average intensity lifetime of ATTO647N-DNA for all three constructs (monothiol-T30, monothiol-psT30, and dithiol-psT30) when bound to NP (blue) or off NP (orange). Intact SNAs were measured at 5 nM (AuNP concentration) and free DNA samples were measured at 50 nM in 1X PBS at RT. **B.** Plot showing the average intensity lifetime for a titration of unbound ATTO647N-DNA (0-10 nM) to 0.5 nM DNA-AuNP in 1X PBS. The number of fluorescent DNA strands on a single nanoparticle is approximately 12, assuming each AuNP presents ~120 nucleotides and 10% are fluorescently labeled on average. Hence, the 6 nM of added ATTO647-DNA is at similar nucleic acid concentration to that of the 0.5 nM AuNP. Data was fit to a sigmoidal function. The positive control (+) is ATTO647N-DNA without AuNP. Statistics were conducted using student t-tests with P values reported as **** ($P < 0.0001$). Experiments were measured in triplicate at RT. Note that only 10% of nucleic acids on the AuNP are fluorescently labeled (**Figure S8**).

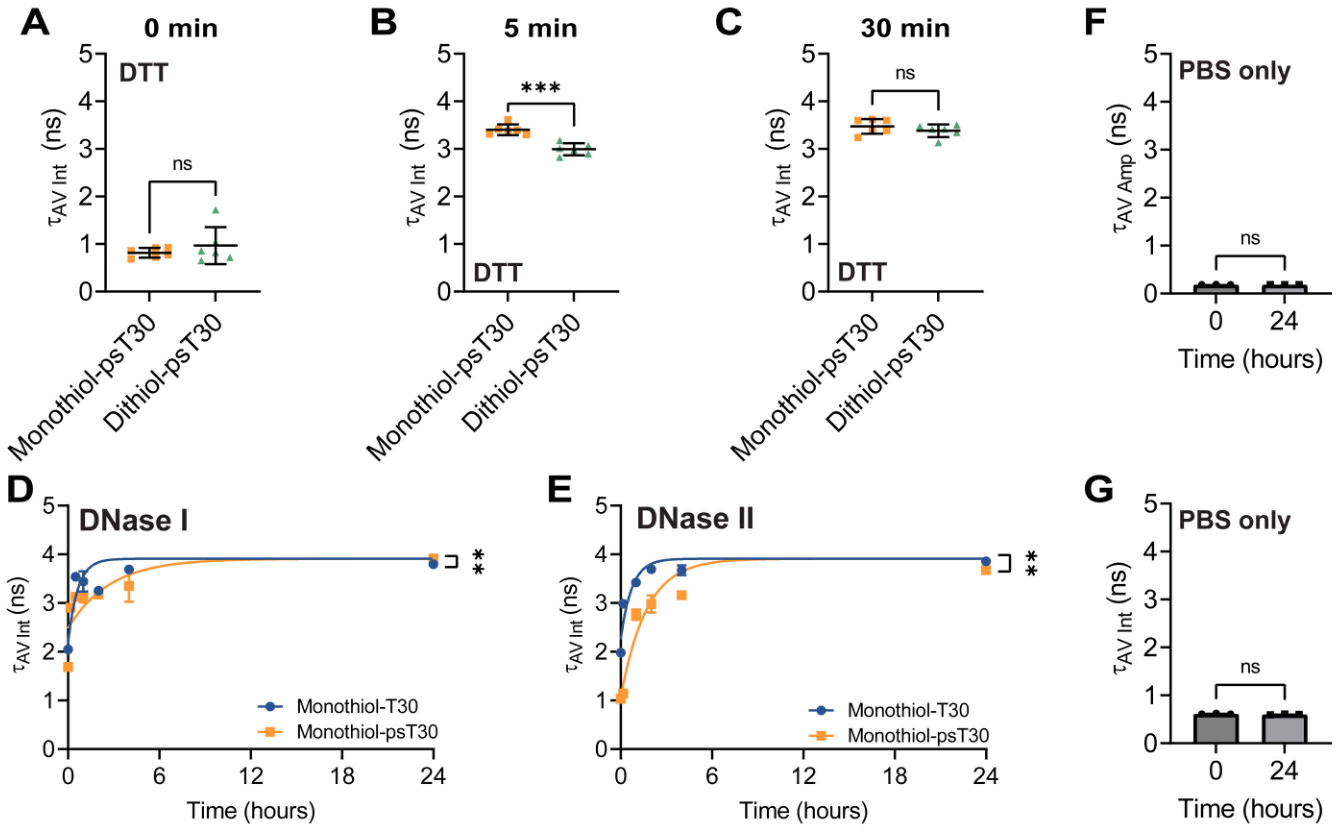

Figure S3: Intensity-weighted FLIM measurements of SNA stability against reducing agents and nucleases. A-C. Plots showing the intensity-weighted lifetime for 0.5 nM monothiol-psT30 and dithiol-psT30 SNA constructs after 0-minute (**A**), 5-minute (**B**), and 30-minute (**C**) incubations with 100 µM DTT at RT in 1X PBS. **D.** Plot showing average intensity lifetimes for 0.5 nM monothiol-T30 and monothiol-psT30 SNA constructs after treatment with 5U DNase I over a 24-hour period in DNase I optimized buffer (10 mM Tris-HCl, 2.5 mM $MgCl_2$, 0.1 mM $CaCl_2$, pH 7.5). **E.** Plot showing average intensity lifetimes for 0.5 nM monothiol-T30 and monothiol-psT30 SNA constructs after treatment with 5U DNase II over a 24-hour period in DNase II optimized buffer (1x UB4 buffer, 117 mM NaCl, pH 5.0). **F-G.** Plots showing amplitude-weighted lifetimes (**F**) or intensity-weighted lifetimes (**G**) for 0.5 nM monothiol-T30 SNA before and after 24h incubation in 1X PBS at RT. Statistics were conducted using an unpaired student's t-test (**A-C, F-G**) or extra sum-of-squares F test (**D**, **E**) with P values reported as ns ($P > 0.05$), ** ($P < 0.01$), and *** ($P < 0.001$). Measurements were conducted in at least triplicate at RT.

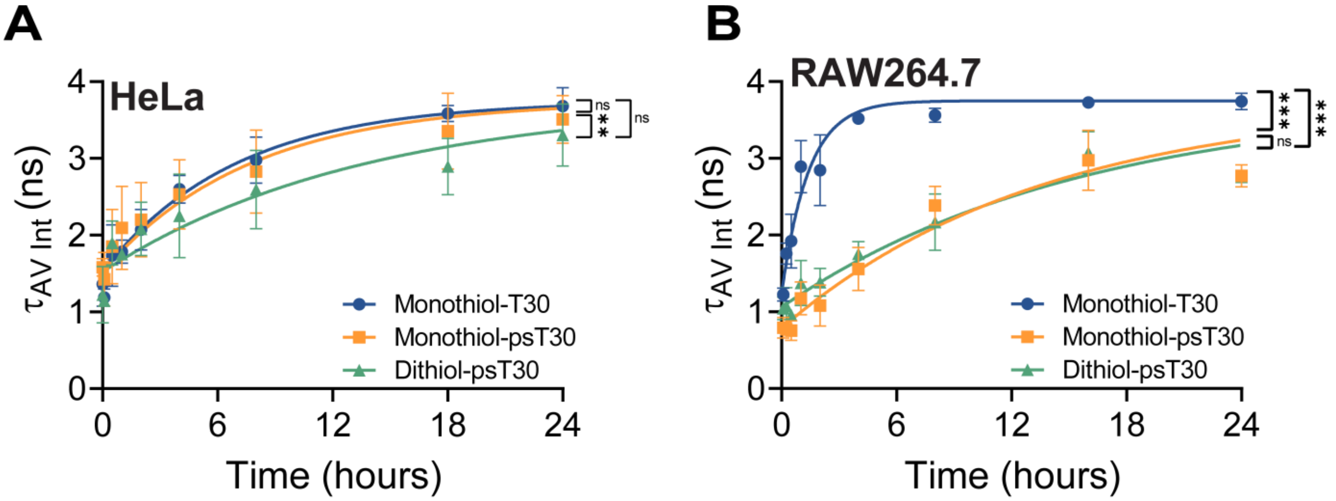

Figure S4: Quantification of SNA dissociation in cells by intensity-weighted FLIM measurements. A. Plot showing the average intensity-weighted lifetimes of monothiol-T30, monothiol-psT30, and dithiol-psT30 SNAs across a 24h timelapse in live HeLa cells. Cells were treated with 5 nM SNA for 5 min, and then the media was washed twice to remove soluble SNAs and synchronize the entry time. **B.** Plot showing the average intensity-weighted lifetimes of monothiol-T30, monothiol-psT30, and dithiol-psT30 SNAs across a 24h timelapse in live RAW264.7 cells. Cells were pulsed with 5 nM SNA for five minutes and left to incubate up to 24h before conducting FLIM measurements without fixation. Lifetimes were quantified using a biexponential reconvolution model. Data was fit using a one-phase association model with the plateau constrained to the free ATTO647N-DNA lifetime in solution (τ_{av} int = 3.75 n.s.). Repeated measures one-way ANOVA tests were conducted to determine significance with post-hoc Tukey's tests upon significance. P values are reported as ns ($P > 0.05$), ** ($P < 0.01$), and *** ($P < 0.001$). All measurements were conducted in at least biological triplicates with multiple cells collected for each data point as technical replicates. Cells were grown in 5% CO_2, 100% humidity, and 37 °C.

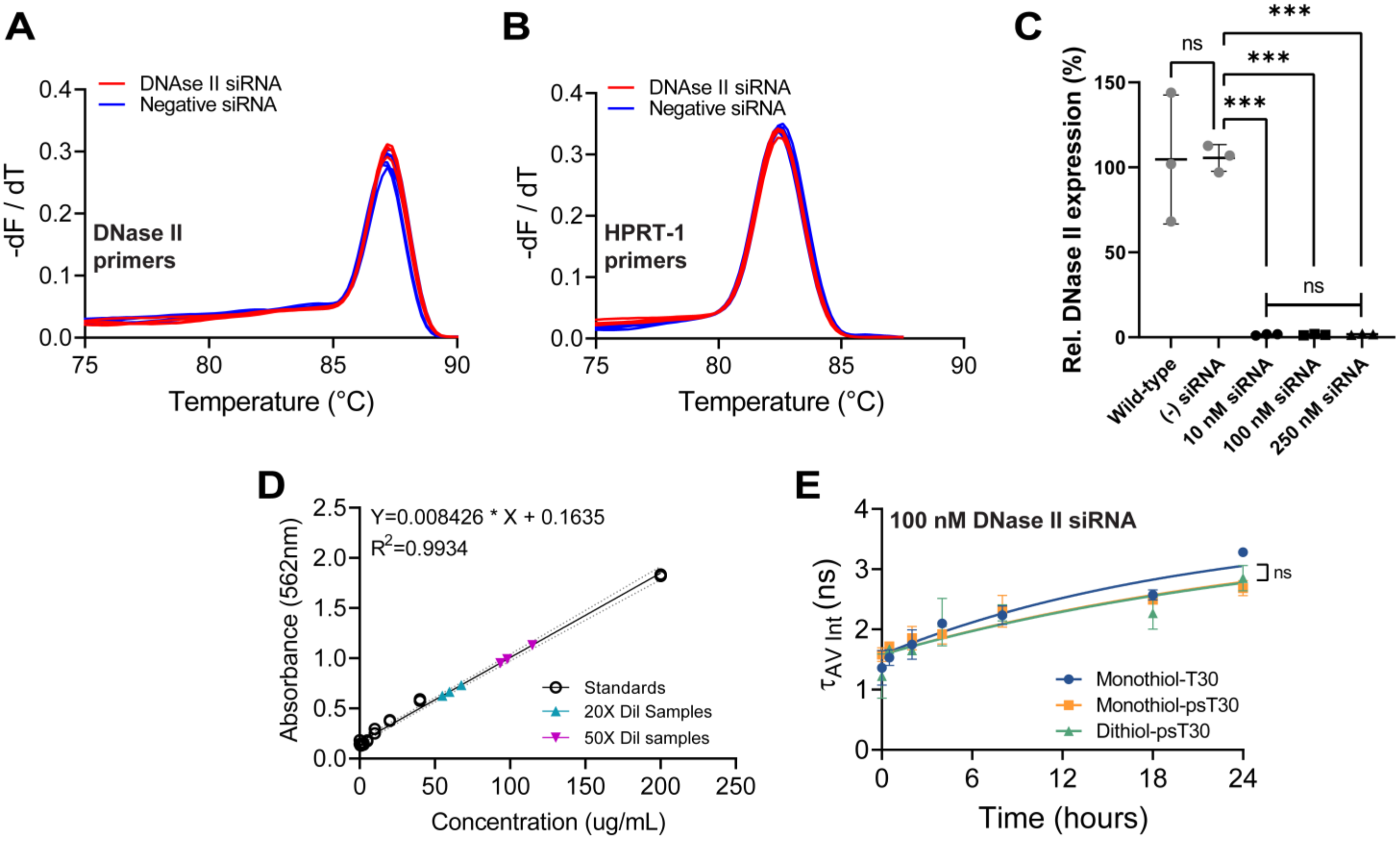

Figure S5: Validation of siRNA-mediated knockdown of DNase II in HeLa cells. A-B. Plots showing high resolution melting analysis of DNase II (**A**) and HPRT-1 (**B**) amplicons after RT-qPCR with primers targeting each respective sequence. HeLa cells were transfected with 100 nM DNase II siRNA (red) or 100 nM negative control siRNA (blue) using Oligofectamine for 24h. **C.** Quantification of relative DNase II expression for wild-type, 100 nM negative control siRNA-treated, and 10-250 nM DNase II siRNA-treated HeLa cells. Relative DNase II expression is determined through the RT-qPCR ΔΔCt method with the nontreated wild-type group as 100% expression and HPRT-1 as the housekeeping gene. **D.** Plot showing results of BCA assay to interpolate total protein concentration from HeLa cell lysates. Cells were either left untreated, treated with 100 nM DNase II siRNA, or 100 nM negative control siRNA and incubated for 48h. The 95% confidence interval is shown by dotted lines with two technical replicates (20x diluted, teal or 50x diluted, purple) for each sample. **E.** Plot showing the average intensity-weighted lifetimes of monothiol-T30, monothiol-psT30, and dithiol-psT30 SNA constructs in HeLa cells treated with 100 nM DNase II siRNA. Cells were treated with 5 nM SNAs for five minutes and left to incubate up to 24h. Data was fit using a one-phase association model. Statistical analysis was conducted using a one-way ANOVA with post-hoc Tukey's tests (**C**) or an extra sum-of-squares F test (**E**) with P values reported as ns ($P > 0.05$) and *** ($P < 0.001$). All measurements were conducted in at least biological triplicates with multiple cells collected for each data point as technical replicates. Cells were grown in 5% CO_2, 100% humidity, and 37 °C.

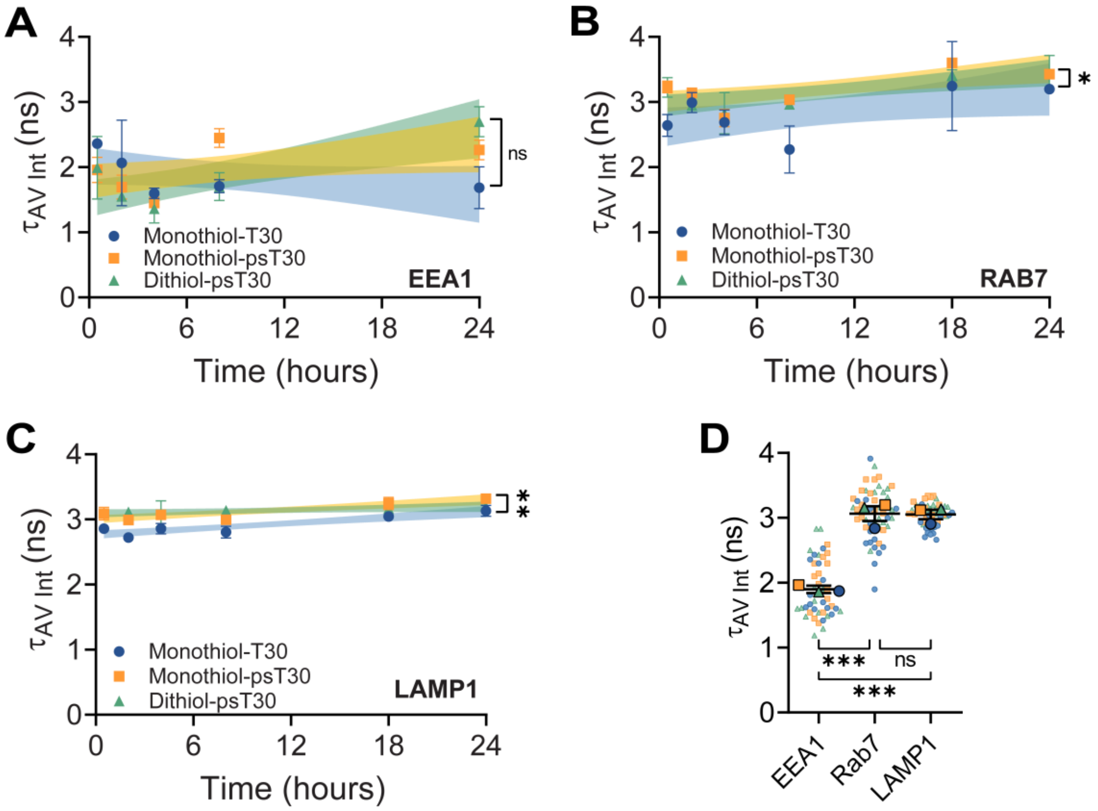

Figure S6: Endosomal staining intensity-weighted lifetime analysis of SNA constructs. A-C. Plots showing the average intensity-weighted lifetimes of all three SNA constructs (monothiol-T30 in blue, monothiol-psT30 in yellow, and dithiol-psT30 in green) for EEA1 (**A**), Rab7 (**B**), and LAMP1 (**C**) staining over a 24h timeframe. Data are fit using a linear regression with the 95% confidence interval shown for each SNA construct by the colored band. **D.** Superplot showing the average intensity lifetimes for EEA1, Rab7, and LAMP1 staining in HeLa cells with all timepoints grouped together. Monothiol-T30 (blue circles), monothiol-psT30 (yellow squares), and dithiol-psT30 (green triangle) are shown with the large shapes representing the mean across the 24h timelapse and smaller shapes representing each individual datapoint. For **A-D**, statistics were conducted using a repeated measures one-way ANOVA with post-hoc Tukey's tests upon significance. P values are reported as ns ($P > 0.05$), * ($P < 0.05$), ** ($P < 0.01$), and *** ($P < 0.001$). For **D**, statistical comparisons were conducted using each SNA mean (n=3) rather than individual data points (n > 40). Cells were grown in 5% CO_2, 100% humidity, and 37 °C. All experiments were conducted in biological triplicate.

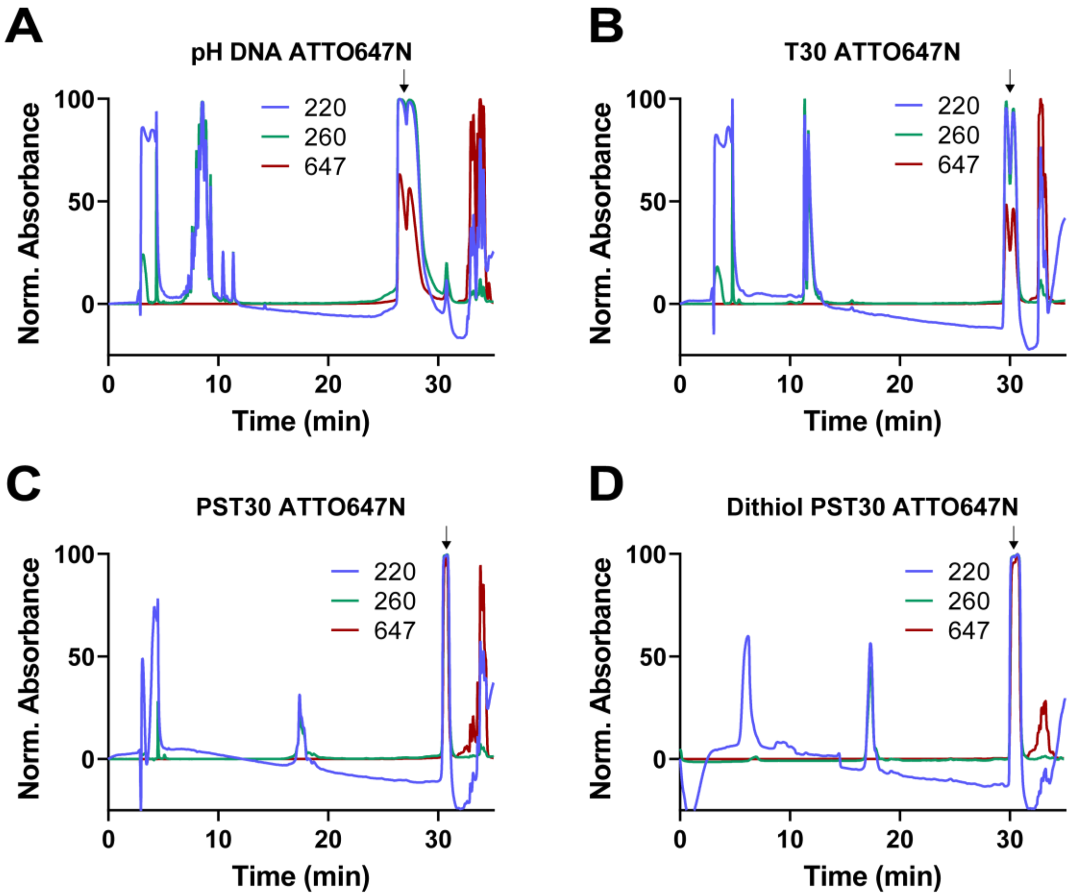

Figure S7: HPLC purification of ATTO647N-labeled oligonucleotides. Chromatograms showing the 220 nm, 260 nm, and 647 nm absorbance measurements for (**A**) pH DNA, (**B**) monothiol T30 DNA, (**C**) monothiol PST30 DNA, and (**D**) dithiol PST30 DNA. The arrow indicates the product peak that was collected and used for experiments throughout the manuscript. HPLC was performed as described within the methods section in the main text.

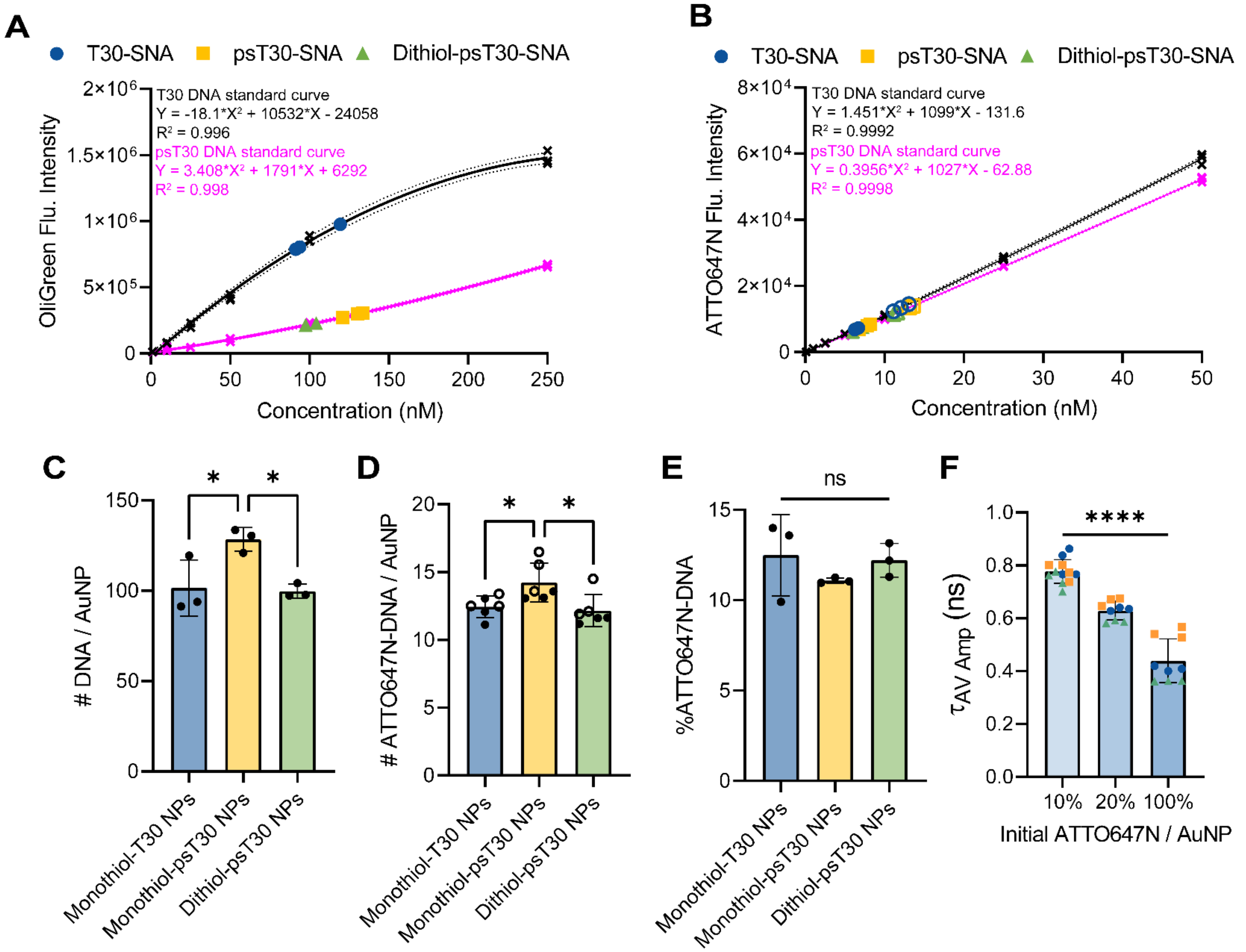

Figure S8: Characterization of DNA loading on DNA-AuNP constructs. A. Plot showing the standard curve for the OliGreen ssDNA assay. Concentrated solutions containing a mixture of 10:1 unlabeled DNA:ATTO647N-DNA was serially diluted from 250 nM (225 nM unlabeled DNA, 25 ATTO647N-DNA) to 1 nM (0.9 nM unlabeled DNA, 0.1 ATTO647N-DNA) total DNA for PO T30 DNA and PS T30 DNA. The specific concentrations were 250 nM, 100 nM, 50 nM, 25 nM, 10 nM, and 1 nM total DNA. Note: OliGreen exhibits varying fluorescence depending on backbone modification which is why two standard curves were prepared. DNA-AuNP constructs were plated at 1 nM AuNP concentration in 100 µL of 1X TE buffer and 1 µL of 1 M KCN solution was added to etch AuNPs for 30min at RT. After incubation, an equal volume solution containing a 50x dilution of OliGreen reagent in 1X TE buffer was added and OliGreen fluorescence intensity was measured using a platereader (480nm excitation, 520nm emission). **B.** Plot showing the ATTO647N-DNA standard curve to determine ATTO647N-labeling for DNA-AuNP constructs. Samples were prepared as above, with ATTO647N fluorescence measured in place of OliGreen fluorescence. Samples were plated at both 0.5 nM (solid shapes) and 1 nM (hollow shapes) AuNP concentrations. The ATTO647N standard concentrations were 50 nM, 25 nM, 10 nM, 5 nM, 2.5 nM, 1 nM, and 0.1 nM. For **A-B**, standards were fit using a quadratic function and sample concentration was interpolated from measured fluorescence. The 95% CI is shown by dotted lines. **C.** Plot showing the total # DNA per AuNP for all three constructs. Three batches were prepared for each DNA-AuNP construct (monothiol-T30, monothiol-psT30, and dithiol-psT30 SNAs) using the freeze method as described in the main text (10:1 unlabeled DNA to ATTO647N-DNA). **D.** Plot showing the # ATTO647N-DNA per AuNP for all three constructs. **E.** Plot showing the measured percentage labeling for ATTO647N-DNA to total DNA per AuNP for each DNA-AuNP construct. **F.** Plot showing the amplitude-weighted lifetime for solution measurements of intact DNA-AuNP constructs prepared as 10%, 20%, and 100% ATTO647N-DNA to study homoFRET interactions. Individual points are colored based on DNA sequence. For **C-F**, statistics were conducted using an ordinary one-way ANOVA with post-hoc Tukey's tests upon significance. P values are reported as ns ($P > 0.05$), * ($P < 0.05$), and **** ($P < 0.0001$).

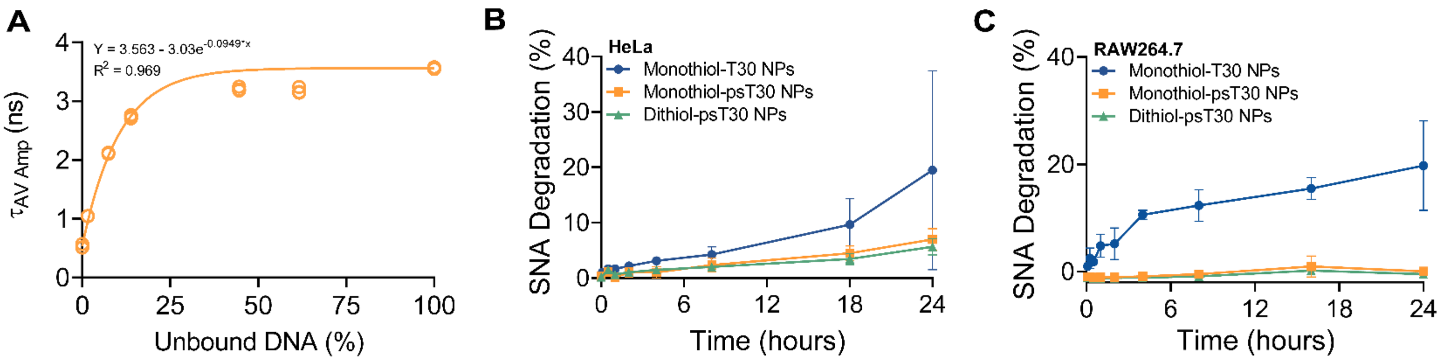

Figure S9: Evaluation of the percentage degradation of DNA-AuNP constructs in cells. A. Plot showing the average amplitude lifetime as a function of %unbound DNA from the titration experiment (**Fig. 1E**). Note: unbound DNA % is determined by the DNA molar ratio between unbound DNA and DNA bound to the AuNP (0.5 nM AuNP, ~6 nM DNA-ATTO647N). Data was fit to a one-phase exponential association function with the equation and goodness of fit shown. **B-C.** Plots showing the SNA degradation% for the (**B**) HeLa cell and (**C**) RAW264.7 macrophages timelapse experiment (data from **Fig. 3D,E**). Using the exponential equation (**A**), %SNA degradation was calculated from the amplitude-weighted lifetime timelapses.

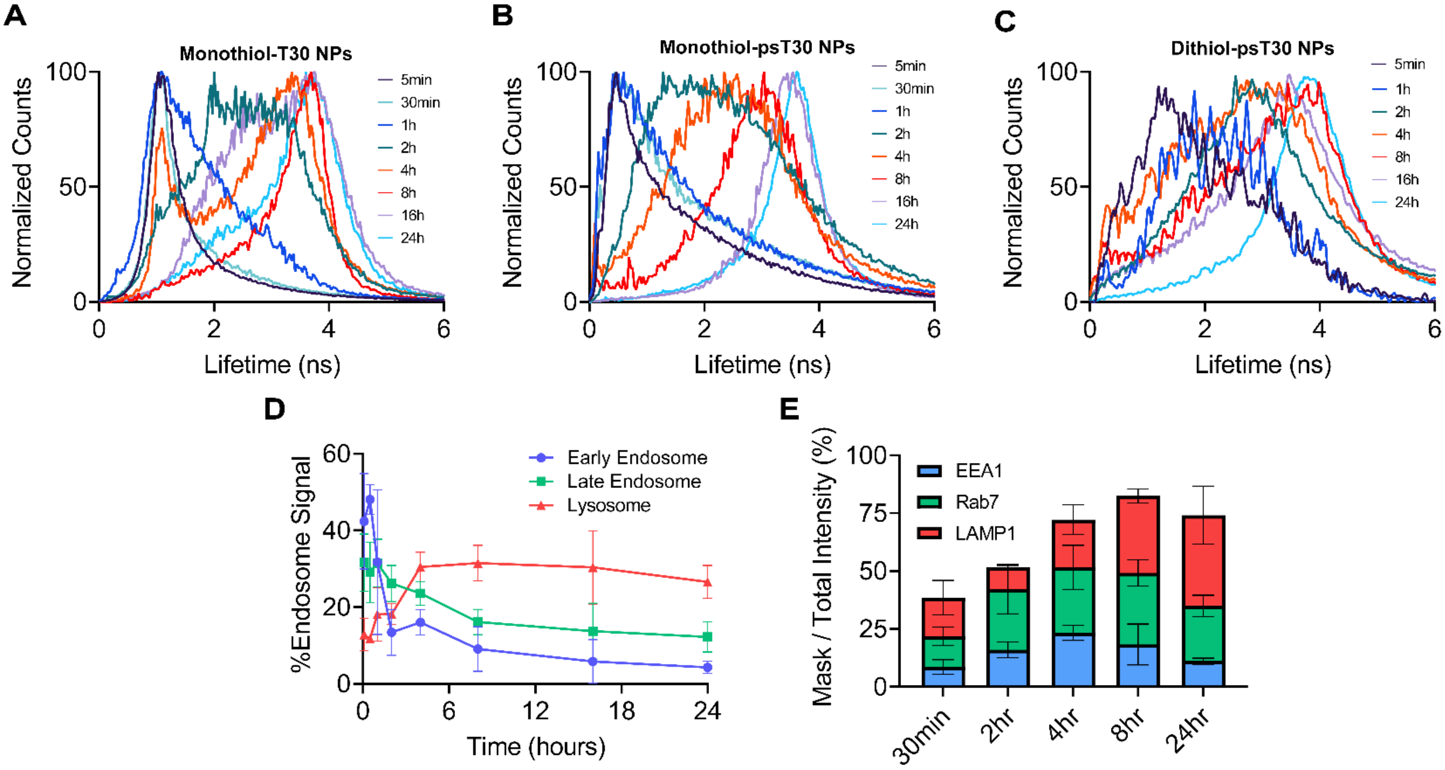

Figure S10: Evaluation of endosomal entrapment in HeLa cells. A-C. Histograms showing the distribution of lifetimes for (**A**) Monothiol-T30 NPs, (**B**) Monothiol-psT30 NPs, and (**C**) Dithiol-psT30 NPs in HeLa cells across a 24h timelapse (Data from **Fig. 3D**). Histograms signal is normalized so that the highest count per timepoint is 100%. **D.** Plot showing the % of endosomal/lysosomal associated fluorescence events as a function of time. The histograms shown in **A**, **B**, and **C** were gated using the lifetime values determined in **Figure 5I** for early and late endosomes as well as lysosomes. Early endosomes had a lifetime of 0.0 -1.2 ns, late endosomes were 1.2-2.0 ns and lysosomes had lifetimes of 2.0-2.6 ns. The fraction of events assigned as early/late endosomes and lysosomes are plotted as blue, green, and red. Each data point represents triplicate measurements with DNA-AuNP constructs combined for each timepoint. **E.** Stacked plot showing the percentage of masked signal over total cell intensity across a 24h timelapse (Data from **Fig 5J-L**). The plot in **E** does not employ the lifetime measurements and the analysis is performed colocalizing the nucleic acid signal with the specific antibody markers of early and late endosomes as well as lysosomes.

Monothiol-T30-ATTO647N

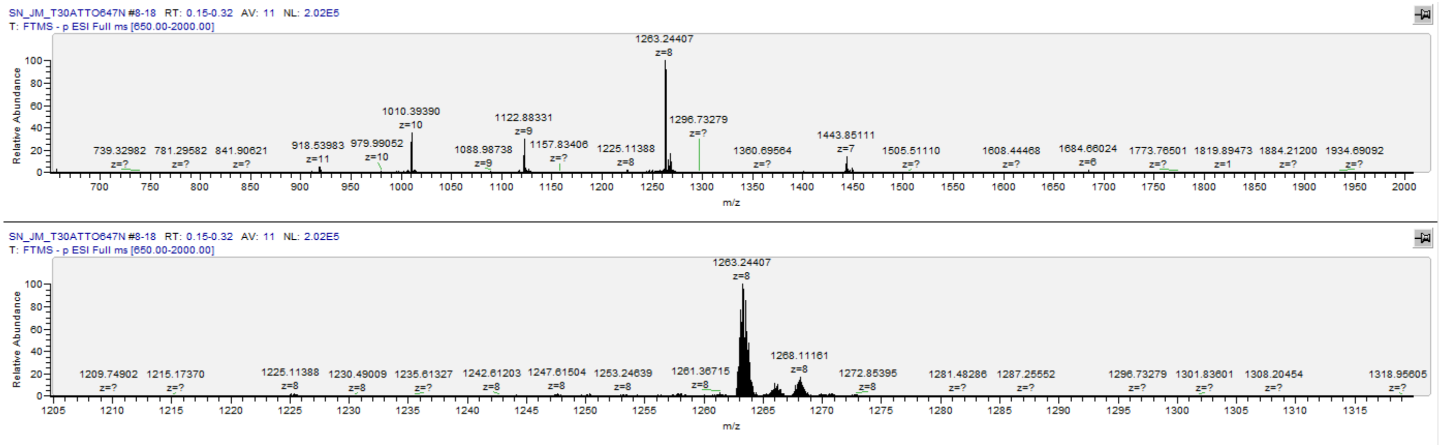

Monothiol-psT30-ATTO647N

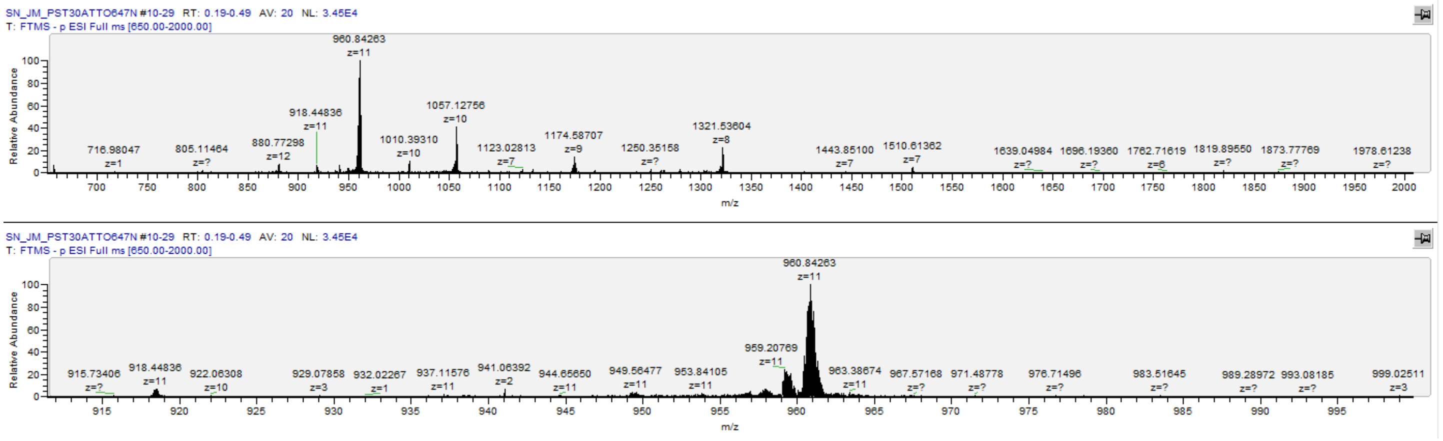

Dithiol-psT30-ATTO647N

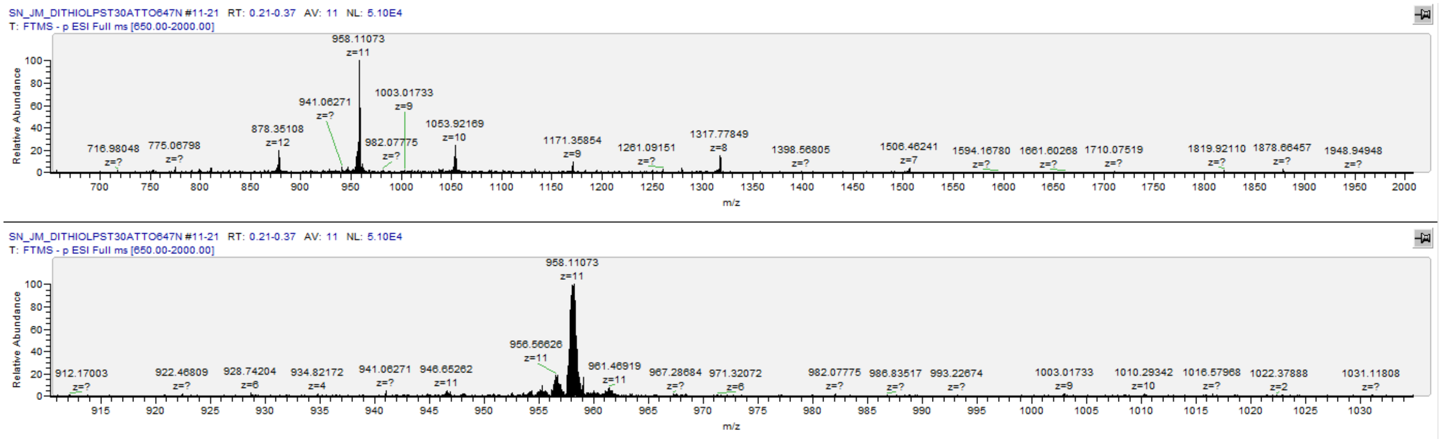

Figure S11: Electrospray Ionization Mass spectroscopy (ESI-MS) characterization of purified oligos. ATTO647N NHS-ester dye was reacted with amine-modified DNA as described in the main text. High resolution mass spectra are shown along with the respective sample name. **Supporting table 2** contains the calculated masses from these data.

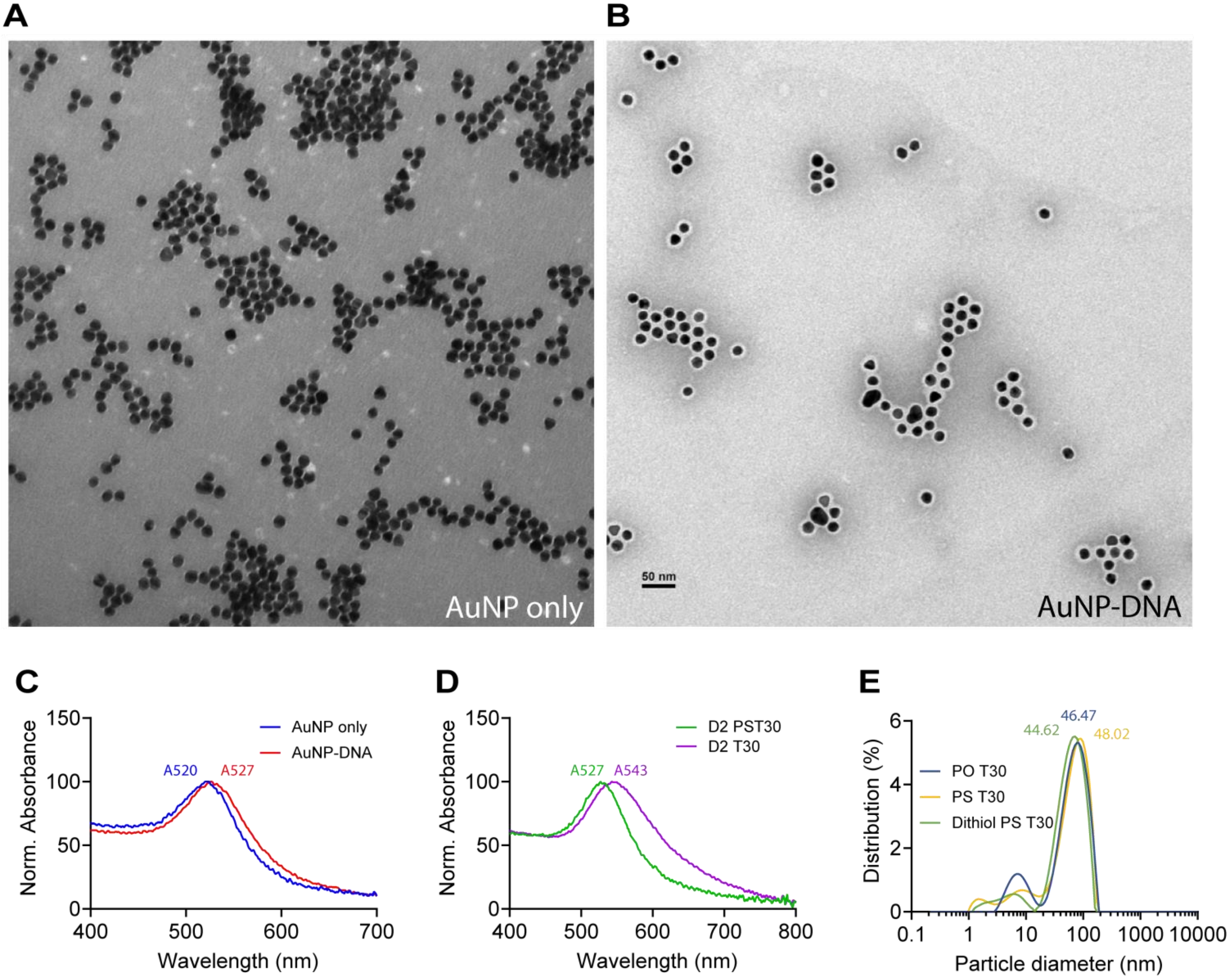

Figure S12: Absorbance characterization of SNA. A-B. Transmission Electron Microscopy (TEM) image of synthesized AuNPs without DNA (**A**) and functionalized with T30 DNA (**B**). A drop of AuNPs were added to a plasmon etched 400-mesh copper grid (~30s) before being dried and imaged. Scale bar = 50nm. **C.** Plot showing normalized absorbance measurements for 15-nm AuNP functionalized without DNA (blue) or with DNA (red, T30 DNA). AuNPs were functionalized as described in the main text using the freeze method. A red shift is visible indicating DNA attachment. **D.** Plot showing normalized absorbance measurements for each SNA construct following a 3h treatment with 5U DNase 2 (D2). The absorption peaks are labeled in the respective colors of each construct. **E.** Plot showing the starting SNA construct particle size as measured through DLS. All particles are similar in size with hydrodynamic diameter labeled and colored.

REFERENCES

(1) Sharma, R.; Dong, Y.; Hu, Y.; Ma, V. P.; Salaita, K. Gene Regulation Using Nanodiscs Modified with HIF-1-alpha Antisense Oligonucleotides. *Bioconjug Chem* **2022**, *33* (2), 279-293. DOI: 10.1021/acs.bioconjchem.1c00505.

(2) Sharma, R.; Narum, S.; Liu, S.; Dong, Y.; Baek, K. I.; Jo, H.; Salaita, K. Nanodiscoidal Nucleic Acids for Gene Regulation. *ACS Chem Biol* **2023**, *18* (11), 2349-2367. DOI: 10.1021/acschembio.3c00038.

(3) Guan, C.; Chernyak, N.; Dominguez, D.; Cole, L.; Zhang, B.; Mirkin, C. A. RNA-Based Immunostimulatory Liposomal Spherical Nucleic Acids as Potent TLR7/8 Modulators. *Small* **2019**, *15* (43), e1903338. DOI: 10.1002/smll.201903338.

(4) Radovic-Moreno, A. F.; Chernyak, N.; Mader, C. C.; Nallagatla, S.; Kang, R. S.; Hao, L.; Walker, D. A.; Halo, T. L.; Merkel, T. J.; Rische, C. H.; et al. Immunomodulatory spherical nucleic acids. *Proceedings of the National Academy of Sciences* **2015**, *112* (13), 3892-3897. DOI: doi:10.1073/pnas.1502850112.

(5) Huang, Z.; Ma, X.; Jiang, F.; Wang, R.; Wu, Z.; Lu, Y. Dual Spatially Localized DNA Walker for Fast and Efficient RNA Detection. *Nano Lett* **2023**, *23* (13), 6042-6049. DOI: 10.1021/acs.nanolett.3c01349 From NLM Medline.

(6) Feng, X.; Bi, X.; Feng, J.; Hu, S.; Wang, Y.; Zhao, S.; Zhang, L. Proximity-Induced Bipedal DNA Walker for Accurately Visualizing microRNA in Living Cancer Cell. *Anal Chem* **2024**, *96* (26), 10669-10676. DOI: 10.1021/acs.analchem.4c01483 From NLM Medline.

(7) Petree, J. R.; Yehl, K.; Galior, K.; Glazier, R.; Deal, B.; Salaita, K. Site-Selective RNA Splicing Nanozyme: DNAzyme and RtcB Conjugates on a Gold Nanoparticle. *ACS Chemical Biology* **2018**, *13* (1), 215-224. DOI: 10.1021/acschembio.7b00437.

(8) Wei, H.; Wang, E. Nanomaterials with enzyme-like characteristics (nanozymes): next-generation artificial enzymes. *Chem Soc Rev* **2013**, *42* (14), 6060-6093. DOI: 10.1039/c3cs35486e.

(9) Wu, P.; Hwang, K.; Lan, T.; Lu, Y. A DNAzyme-gold nanoparticle probe for uranyl ion in living cells. *J Am Chem Soc* **2013**, *135* (14), 5254-5257. DOI: 10.1021/ja400150v From NLM Medline.

(10) Pitou, M.; Papi, R. M.; Tzavellas, A.-N.; Choli-Papadopoulou, T. ssDNA-Modified Gold Nanoparticles as a Tool to Detect miRNA Biomarkers in Osteoarthritis. *ACS Omega* **2023**, *8* (8), 7529-7535. DOI: 10.1021/acsomega.2c04806.

(11) Daniel, W. L.; Lorch, U.; Mix, S.; Bexon, A. S. A first-in-human phase 1 study of cavrotolimod, a TLR9 agonist spherical nucleic acid, in healthy participants: Evidence of immune activation. *Front Immunol* **2022**, *13*, 1073777. DOI: 10.3389/fimmu.2022.1073777 From NLM Medline.

(12) Lewandowski, K. T.; Thiede, R.; Guido, N.; Daniel, W. L.; Kang, R.; Guerrero-Zayas, M. I.; Seeger, M. A.; Wang, X. Q.; Giljohann, D. A.; Paller, A. S. Topically Delivered Tumor Necrosis Factor-alpha-Targeted Gene Regulation for Psoriasis. *J Invest Dermatol* **2017**, *137* (9), 2027-2030. DOI: 10.1016/j.jid.2017.04.027 From NLM Medline.
